# β-glucuronidase inhibitors isolated from the fruit of *Forsythia suspensa* (Thunb.) Vahl

**DOI:** 10.1007/s11418-026-02070-1

**Published:** 2026-07-28

**Authors:** Shogo Nishikawa, Ryuya Takada, Yusuke Shimomoto, Sara Morishita, Yoshiaki Manse, Tomoe Ohta, Tatsusada Yoshida, Takahiro Matsumoto, Toshio Morikawa

**Affiliations:** 1 Pharmaceutical Research and Technology Institute, Kindai University, 3-4-1 Kowakae, Higashi-osaka, Osaka, 577-8502 Japan; 2 Antiaging Center, Kindai University, 3-4-1 Kowakae, Higashi-osaka, Osaka, 577-8502 Japan; 3 Graduate School of Pharmacy, Kindai University, 3-4-1 Kowakae, Higashi-Osaka, Osaka, 577-8502 Japan; 4 Kyoto Pharmaceutical University, Misasagi, Yamashina-ku, Kyoto, 607-8412 Japan; 5 Faculty of Pharmaceutical Sciences, Nagasaki International University, 2825-7 Huis Ten Bosch Machi, Sasebo, Nagasaki 859-3298 Japan

**Keywords:** *Forsythia suspensa*, Rengyonoic acid, Diterpene, β-Glucuronidase inhibitory activity, Oleaceae

## Abstract

A methanol extract from the fruit of *Forsythia suspensa* (Thunb.) Vahl (Oleaceae), which is used as the crude drug Forsythiae Fructus (*Forsythia Fruit*; “*Rengyo*” in Japanese) and is listed in the Japanese Pharmacopoeia XIX, exerted enzymatic β-glucuronidase inhibitory activity. From the extract, we isolated a new diterpene rengyonoic acid (**1**) along with 19 known compounds including two diterpene (**2** and **3**), three triterpenes (**4**–**6**), seven lignans (**7**–**13**), two flavonoids (**14** and **15**), four phenylethanoids (**16**–**19**), and an aromatic compound (**20**). The structure of the new compound (**1**), including its absolute stereochemistry, was determined based on chemical and physicochemical evidence derived from NMR and MS spectrometry analysis as well as by comparing experimental and predicted ECD data. Among the isolates, **1** (IC_50_ = 117 μM), pinoresinol (**7**, 27.9 μM), forsythiaside A (**16**, 18.6 μM), and suspensaside A (**19**, 31.1 μM) exhibited β-glucuronidase inhibitory activity equivalent to that of a positive compound, d-saccharic 1,4-lactone (38.6 μM).

**Graphical abstract:**

**Figure:**
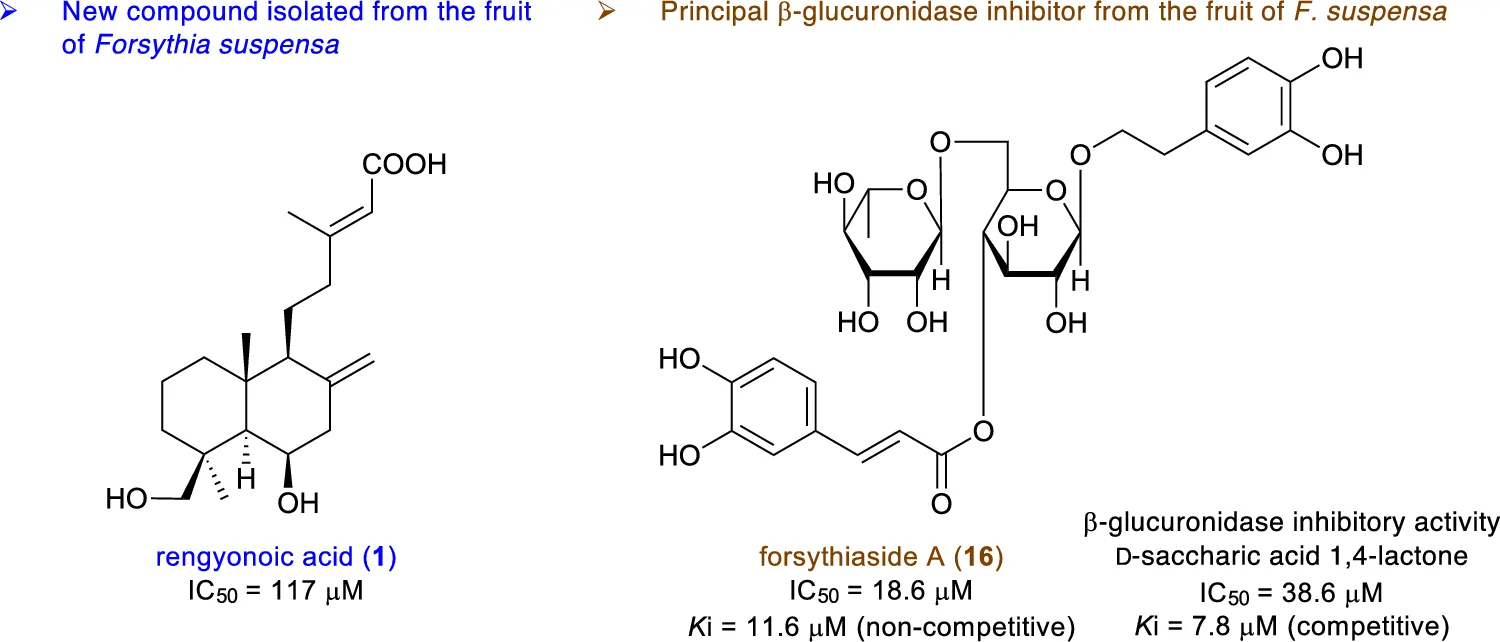

**Supplementary Information:**

The online version contains supplementary material available at https://doi.org/10.1007/s11418-026-02070-1.

## Introduction

The plant *Forsythia suspensa* (Thunb.) Vahl belonging to the Oleaceae family (an accepted name according to The World Flora Online [1]) is an ornamental shrub widely distributed primarily in northern mainland China. The fruit of this plant, Forsythiae Fructus (*Forsythia Fruit*; "*Rengyo*" in Japanese), is listed in the Japanese Pharmacopoeia (JP XIX) and is utilized as a common traditional medicine in China and Korea used to treat inflammation, pyrexia, ulcers, gonorrhea, and erysipelas [2–4]. Previous phytochemical studies of the constituents of the fruit of this plant revealed triterpenes [2, 5–8], lignans [2, 5, 7, 8], flavonoids [2, 5, 7, 8], phenylethanoids [2, 9–15], cyclohexylethane derivatives [2, 16], and alkaloids [2, 17], etc. Recent studies reported several diterpenes obtained from the fruit [2, 17, 18] and the seeds of this plant [3, 4]. The extract and/or its constituents exert anti-inflammatory [2–4, 15, 17–24], vasorelaxant [25], antioxidant [2–4, 17, 26, 27], antibacterial [2–4, 9, 11, 28], antivirus [2–4, 29–31], antitumor [2–4, 32], antiallergy [2, 33], and neuroprotective effects [2–4, 15, 34, 35], etc. While characterizing the active constituents responsible for the medicinal properties of crude drugs listed in the JP, such as Curcuma Rhizome (*Curcumae Rhizoma*; “*Gajutsu*” in Japanese) [36–42], Bitter Cardamon (*Alpiniae Fructus*; “*Yakuchi*”) [43, 44], Alpinia Officinarum Rhizome (*Apliniae Offcinarum Rhizoma*; “*Ryokyou*”) [45], Chrysanthemum Flower (*Chrysanthemi Flos*; “*Kikuka*”) [46–48], Saussurea Root (*Saussureae Radix*; “*Mokkou*”) [49, 50], Magnolia Bark (*Magnoliae Cortex*; “*Kouboku*”) [51], Nuphar Rhizome (*Nupharis Rhizoma*; “*Senkotsu*”) [52], Cistanche Herb (*Cistanchis Herba*; “*Nikujuyou*”) [53–61], Euodia Fruit (*Euodiae Fructus*; “*Gosyuyu*”) [62–64], Cnidium Rhizome (*Cnidii Rhizoma*; “*Senkyu*”) [65], and Sophora Root (*Sophorae Radix*; “*Kujin*”) [66], we found that the methanol extract of the fruit of *F. suspensa* exhibited β-glucuronidase inhibitory activity. In addition, a new diterpene rengyonoic acid (**1**) was isolated from the extract, along with 19 known compounds (**2**–**20**). Here, we describe the isolation and structure determination of **1** as well as the β-glucuronidase inhibitory activity of the isolates.

## Results and discussion

### Effects of the methanol extract and its fractions against Escherichia coli β-glucuronidase

β-Glucuronidase is a critical enzyme involved in the hydrolysis of glucuronide conjugates and is not only found in anaerobic intestinal bacteria, but also in mammalian organs and body fluids. This enzyme maintains homeostasis and significantly influences drug metabolism, detoxification, and enterohepatic circulation. Dysregulated β-glucuronidase activity has been implicated in diverse pathological conditions, including drug-induced toxicity, inflammation, and hormone-dependent cancers. Particularly, microbial β-glucuronidase expressed by gut microbiota can reactivate glucuronidase-conjugated drugs, leading to adverse reactions. Therefore, inhibition of microbial β-glucuronidase has emerged as a potential therapeutic approach for reducing chemotherapy-induced toxicity, gastrointestinal complications, and metabolic disorders. For example, the active metabolite of the chemotherapeutic agent irinotecan, SN-38, is detoxified through glucuronidation and excreted into the gastrointestinal tract. Intestinal bacteria convert the glucuronidated metabolite back to toxic SN-38 using β-glucuronidase, resulting in debilitating diarrhea. Inhibition of β-glucuronidase activity may relieve this effect [67–73]. Furthermore, degradation of microbial β-glucuronidase conjugates by the gut microbiota contributes to urinary odor formation. Urinary odor components, such as phenolic compounds, are generated during urine decomposition; most of these compounds exist as odorless glucuronide conjugates immediately after urination. These glucuronide conjugates are subsequently hydrolyzed by bacterial β-glucuronidase, producing volatile compounds responsible for urinary odor. Therefore, inhibition of gut bacterial β-glucuronidase is expected to suppress urinary odor generation [74, 75].

We examined the effect of the methanol (MeOH) extract of the fruit of *F. suspensa* against β-glucuronidase. As shown in Table 1, the MeOH extract inhibited enzymatic activity (IC_50_ = 33.2 μg/mL). Through bioassay-guided fractionation, the EtOAc-soluble fraction (22.9 μg/mL) and MeOH-eluted fraction (20.9 μg/mL) were identified as the active fractions.

**Table 1:** Effects of the MeOH extract and its fractions from the fruit of *Forsythia suspensa* against *Escherichia coli* β-glucuronidase

Treatment	Inhibition (%)					IC_50_ (μg/mL)
Conc. (*μ*g/mL)	0	10	30	100	300	
MeOH extract	0.0 ± 3.2	32.5 ± 1.3**	45.4 ± 1.7**	69.2 ± 0.4**	82.0 ± 1.7**	33.2
EtOAc-soluble fraction	0.0 ± 3.2	36.8 ± 1.7**	52.5 ± 1.0**	74.7 ± 0.6**	92.0 ± 1.3**	22.9
MeOH-eluted fraction	0.0 ± 3.7	31.5 ± 2.4**	58.7 ± 2.5**	85.9 ± 0.4**	94.3 ± 0.9**	20.9
H_2_O-eluted fraction	0.0 ± 3.7	15.8 ± 2.8**	26.4 ± 4.5**	71.0 ± 1.0**	94.1 ± 0.4**	53.9

Each value represents the mean ± S.E.M (*N* = 5)

Asterisks denote significant differences from the control group, *p < 0.05, **p < 0.01

### Isolation

**Fig. 1:**
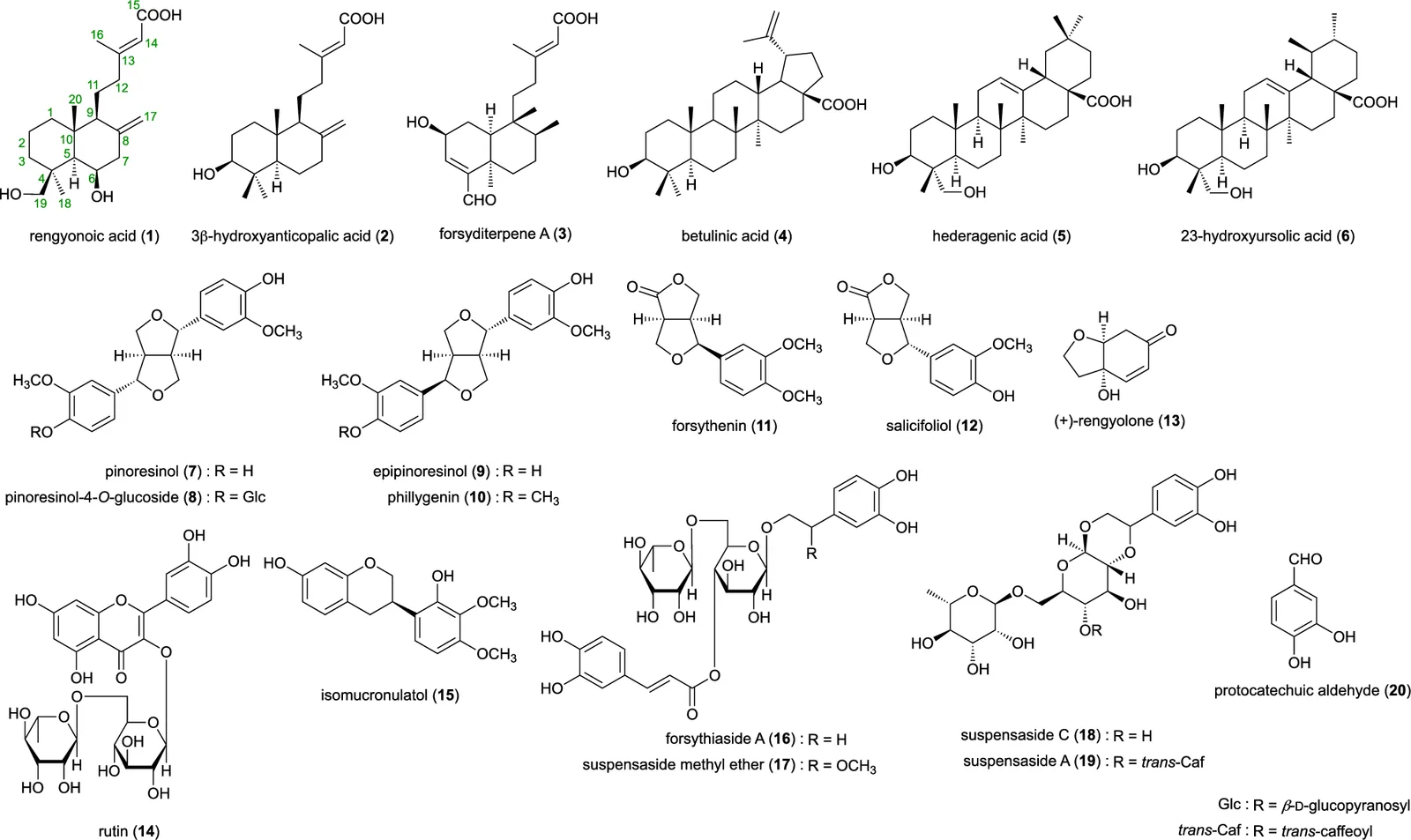
Isolates (**1**–**20**) obtained from the fruit of *Forsythia suspensa*

We isolated a new diterpene rengyonoic acid (**1**, 0.0006%), along with 19 known compounds including two diterpenes, 3β-hydroxyanticopalic acid (**2**, 0.0026%) [76] and forsyditerpene A (**3**, 0.0007%) [3], three triterpenes, betulinic acid (**4**, 0.66%) [77], hederagenic acid (**5**, 0.0011%) [78], and 23-hydroxyursolic acid (**6**, 0.0021%) [79], seven lignans, pinoresinol (**7**, 0.016%) [80], pinoresinol-4-*O*-β-glucoside (**8**, 0.0016%) [81], epipinoresinol (**9**, 0.0038%) [82], phillygenin (**10**, 0.019%) [83], forsythenin (**11**, 0.0006%) [84], salicifoliol (**12**, 0.0003%) [85], and (+)-rengyolone (**13**, 0.00061%) [86], two flavonoids, rutin (**14**, 0.0050%) [87] and isomucronulatol (**15**, 0.00013%) [88], four phenylethanoids, forsythiaside A (**16**, 0.0057%) [87], suspensaside methyl ether (**17**, 0.0007%) [89], and suspensaside C (**18**, 0.0014%) [90] and A (**19**, 0.0063%) [91], and an aromatic compound protocatechuic acid (**20**, 0.0006%) [92] from the MeOH extract using normal-phase silica gel and reversed-phase ODS column chromatography (CC) and finally preparative HPLC as shown in Fig. 1.

### Structure elucidation of rengyonoic acid (*1*)

**Fig. 2:**
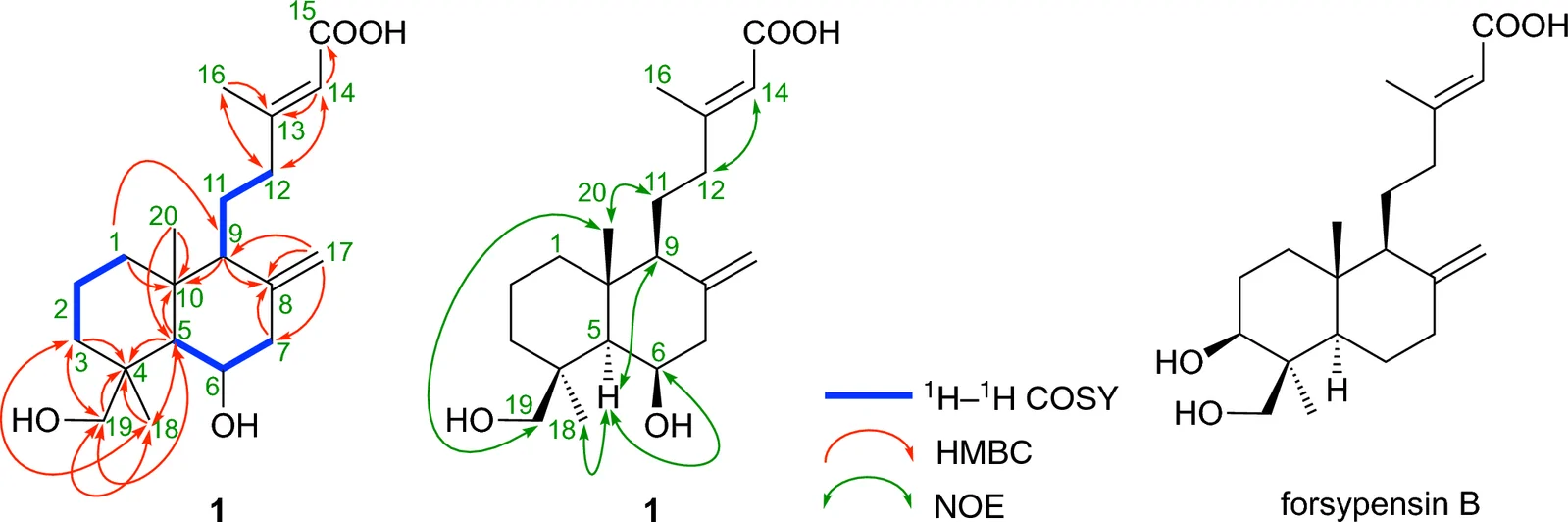
^1^H–^1^H COSY, HMBC, and pNOESY correlations of **1** measured in pyridine-*d*_5_

Rengyonoic acid (**1**) was obtained as a white powder with a positive optical rotation ([*α*]_D_^23^ + 25.2 in MeOH). A quasimolecular ion peak of **1** was observed at *m/z* 359 [M + Na]^+^ in positive-ion electrospray ionization mass spectrometry (ESIMS), and high-resolution ESIMS measurements revealed a molecular formula of C_20_H_32_O_4_. The IR spectrum of **1** showed absorption bands at 3400, 1695, and 1645 cm^−1^, suggesting the presence of hydroxy, conjugated carboxylic acid, and olefinic functionalities. The ^1^H and ^13^C NMR spectroscopic properties (Table 2, pyridine-*d*_5_) of **1**, which were assigned with the aid of distortionless enhancement by polarization transfer (DEPT), ^1^H-^1^H homonuclear correlation (COSY), heteronuclear single quantum coherence (HSQC), and heteronuclear multiple-bond correlation (HMBC) experiments, exhibited signals assignable to three methyls [δ 1.23, 1.33, 2.45 (3H each, all s, H_3_-18, 20, and 16)], eight methylenes including a hydroxy methyl and an exo-methylene {δ [1.10 (1H, ddd, *J* = 3.9, 14.0, 13.1 Hz), 1.75 (1H, m), H_2_-1], [1.20 (1H, ddd, *J* = 4.0, 13.6, 13.7 Hz), 1.57 (1H, m), H_2_-3], [1.42 (1H, dt-like, *J* = *ca.* 4, 14 Hz), 1.61 (1H, ddt-like, *J* = *ca.* 3, 4, 14 Hz), H_2_-2], 1.74, 1.79 (1H each, both m, H_2_-11), 2.14, 2.44 (1H each, both m, H_2_-12), [2.37 (1H, br d, *J* = *ca.* 14 Hz), 2.73 (1H, br dd, *J* = *ca.* 3, 14 Hz), H_2_-7], 3.47, 4.51 (1H each, both d, *J* = 11.4 Hz, H_2_-19), 4.88, 5.14 (1H each, both br s, H_2_-17)}, three methines including a methine bearing an oxygen function [δ 1.32, 1.80 (1H each, both m, H-5 and 9), 4.59 (1H, br s, H-6)], and a tri-substituted olefin proton [δ 6.18 (1H, br s, H-14)]. The labdane-type diterpene skeleton of **1** was determined by the ^1^H-^1^H COSY and HMBC experiments and comparison of the proton and carbon signals with those of related diterpene forsypensin B [93] (Fig. 2). Thus, ^1^H-^1^H COSY experiment indicated the presence of the partial structure shown with bold lines. HMBC experiment of **1** revealed long-range correlations between the following proton and carbon pairs: H_2_-1 and C-9 (δ_C_ 57.1), C-10 (δ_C_ 39.7); H_2_-3 and C-4 (δ_C_ 41.0), C-18 (δ_C_ 28.0), C-19 (δ_C_ 67.6); H-5 and C-4, C-10, C-18, C-19; H_2_-7 and C-8 (δ_C_ 145.1), C-17 (δ_C_ 108.8); H-9 and C-8, C-10; H_2_-12 and C-14 (δ_C_ 117.4), C-16 (δ_C_ 18.6); H-14 and C-13 (δ_C_ 158.9), C-15 (δ_C_ 169.1); H_3_-16 and C-12 (δ_C_ 39.8), C-13; H_2_-17 and C-7 (δ_C_ 46.5), C-8, C-9; H_3_-18 and C-3 (δ_C_ 40.8), C-4, C-5, C-19; H_2_-19 and C-3–5, C-18; and H_3_-20 and C-5, C-10 (Fig. 2). These results clarified the connectivities of the partial structures and quaternary carbons in **1**. In addition, the planar structure of **1** was fully supported by a comparison with the spectral data for forsypensin B [93] measured in the same solvent (DMSO-*d*_6_). Next, the relative stereostructure of **1** was characterized using phase-sensitive nuclear Overhauser enhancement experiment spectroscopy (pNOESY). Although the methyl protons at H_3_-18 and H_3_-20 [δ 0.95 (s)] in **1** were overlapped in DMSO-*d*_6_, they could be clearly distinguished in pyridine-*d*_5_. As shown in Fig. 2, the nuclear Overhauser effect (NOE) correlations of **1** were observed between the following proton pairs: H-5 and H-6, H-9, H_3_-18; H_2_-11 and H_3_-20; H_2_-12 and H-14; H_3_-19 and H_3_-20. On the basis of this evidence, the relative stereostructure of **1** was elucidated.

**Table 2:** ^1^H and ^13^C NMR spectroscopic data of rengyonoic acid (**1**) and forsypensin B

Position	**1** (pyridine-*d*_5_)		**1** (DMSO-*d*_6_)		Forsypensin B (DMSO-*d*_6_)^*a*^	
	*δ*_H_	*δ*_C_	*δ*_H_	*δ*_C_	*δ*_H_	*δ*_C_
1	1.10 (ddd, 3.9, 13.0, 13.1)	41.5	1.02 (ddd, 3.7, 13.1, 13.6)	41.1	1.14 (dd, 2.3, 12.6)	37.0
	1.75 (m)		1.69 (ddd, 3.2, 4.8, 13.6)		1.73 (m)	
2	1.42 (dt-like, *ca.* 4, 14)	19.5	1.37 (m)	19.0	1.61 (2H, m)	28.4
	1.61 (ddt-like, *ca.*3, 4, 14)		1.49 (m)			
3	1.20 (ddd, 4.0, 13.6, 13.7)	40.8	1.06 (m)	39.6	3.23 (dd, 2.5, 10.9)	79.0
	1.57 (m)		1.51 (m)			
4		41.0		40.4		42.8
5	1.32 (m)	58.3	1.20 (d, 1.6)	57.6	1.12 (dd, 2.5, 10.9)	55.2
6	4.59 (br s)	67.3	4.18 (ddd, 1.6, 3.0, 3.2)	66.6	1.33 (m)	24.7
					1.70 (m)	
7	2.37 (br d, *ca.* 14)	46.5	2.15 (dd, 3.2, 13.8)	46.0	1.83 (m)	38.5
	2.73 (br dd, *ca.* 3, 14)		2.31 (dd, 3.2, 13.8)		2.33 (m)	
8		145.1		144.6		148.1
9	1.80 (m)	57.1	1.60 (m)	56.4	1.51 (m)	55.8
10		39.7		39.2		39.3
11	1.74 (m)	22.1	1.52 (m)	21.5	1.45 (m)	21.9
	1.79 (m)		1.59 (m)		1.60 (m)	
12	2.14 (m)	39.8	1.94 (m)	39.2	1.90 (m)	39.6
	2.44 (m)		2.21 (m)		2.20 (m)	
13		158.9		159.1		159.7
14	6.18 (br s)	117.4	5.53 (s)	116.1	5.53 (br s)	116.4
15		169.1		167.5		168.0
16	2.45 (3H, s)	18.6	2.06 (3H, d, 0.8)	18.7	2.06 (3H, s)	18.8
17	4.88 (br s)	108.8	4.63 (br s)	108.5	4.49 (s)	107.1
	5.14 (br s)		4.84 (br s)		4.83 (s)	
18	1.23 (3H, s)	28.0	0.95 (3H, s)	27.7	1.09 (3H, s)	23.6
19	3.47 (d, 11.4)	67.6	3.14 (d, 11.2)	66.1	3.21 (d, 11.0)	63.2
	4.51 (d, 11.4)		3.98 (d, 11.2)		3.82 (d, 11.0)	
20	1.33 (3H, s)	17.1	0.95 (3H, s)	16.9	0.60 (3H, s)	15.4

Measured by 800 MHz for ^1^H-NMR and 200 MHz for ^13^C-NMR

Ref. [93]

**Fig. 3:**
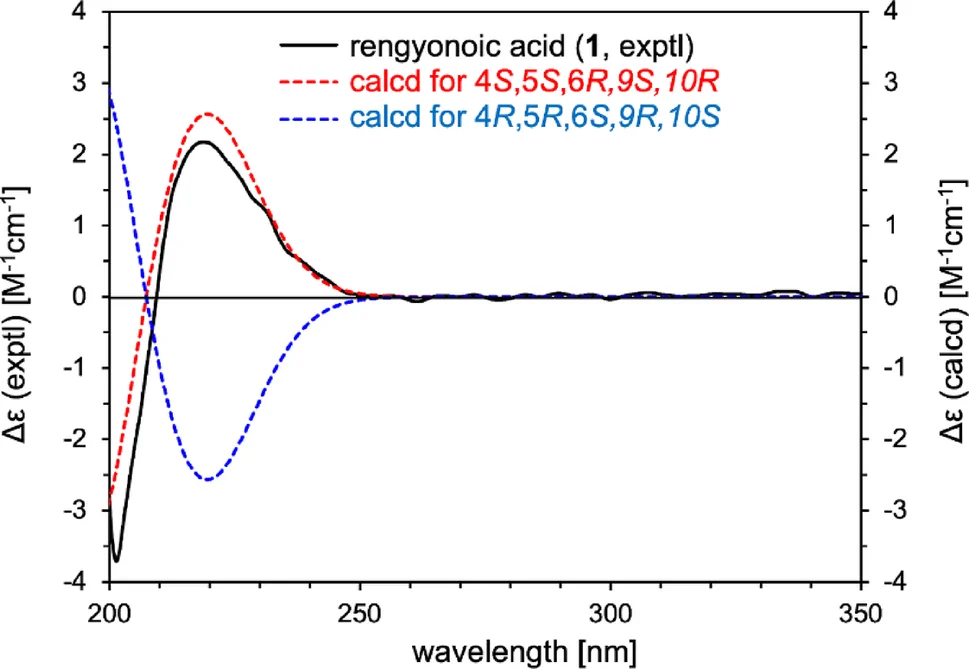
Comparison of the experimental ECD spectrum of **1** with the calculated curves of their conceivable enantiomers. The experimental and calculated curves of **1** (4*S*,5*S*,6*R,*9*S,*10*R*) with calculated curves of the enantiomer (4*R*,5*R*,6*S,*9*R,*10*S*)

**Fig. 4:**
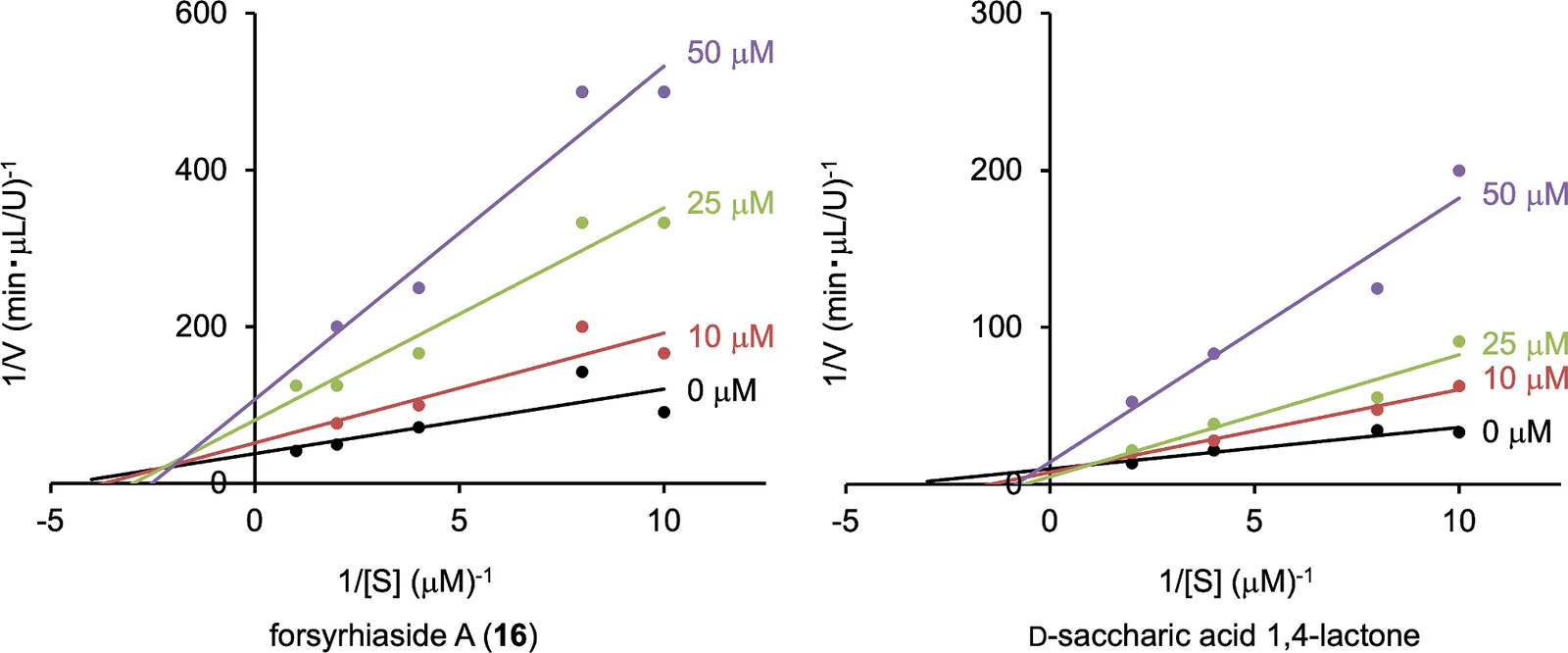
Lineweaver–Burk plots of the Inhibition against *Escherichia coli* β-glucuronidase activities by **16** and d-saccharic acid 1,4-lactone

To determine the absolute stereostructure of **1**, the experimental electronic circular dichroism (ECD) spectrum of **1** [219 nm (Δε + 2.17) in MeOH] was compared with the calculated curves of its conceivable enantiomers. As shown in Fig. 3, (4*S*,5*S*,6*R*,9*S*,10*R*)-form was identical to that of the experimental data obtained for **1**, whereas the calculated data of the *ent*-**1** [(4*R*,5*R*,6*S*,9*R*,10*S*)-form] exhibited an opposite curve. These data suggest that the absolute stereostructure of rengyonoic acid was determined to be (4*S*,5*S*,6*R*,9*S*,10*R*)-6,19-dihydroxylabda-8(17),13(*E*)-diene-15-oic acid (**1**).

### Effects of the isolates against E. coli β-glucuronidase

As shown in Table 3, several isolates such as rengyonoic acid (**1**, IC_50_ = 117 μM), pinoresinol (**7**, 27.9 μM), forsythiaside A (**16**, 18.6 μM), and suspensaside A (**19**, 31.1 μM) inhibited *E. coli* β-glucuronidase, which exhibited the equivalent or relatively strong inhibitory activities as the positive control d-saccharic acid 1,4-lactone (38.6 μM) [70, 94, 95].

**Table 3:** Effects of the isolates from the fruit of *Forsythia suspensa* against *Escherichia coli* β-glucuronidase

Treatment	Inhibition (%)					IC_50_ (μM)
Conc. (μM)	0	10	30	100	300	
Rengyonoic acid (**1**)	0.0 ± 4.7	19.9 ± 0.7**	20.7 ± 1.6**	47.2 ± 3.2**	69.0 ± 1.1**	117
3β-Hydroxyanticopalic acid (**2**)	0.0 ± 3.4	14.9 ± 4.1*	18.0 ± 4.1**	30.0 ± 2.3**	48.7 ± 2.2**	
Forsyditerpene A (**3**)	0.0 ± 4.7	16.2 ± 1.1	16.7 ± 1.2	29.2 ± 2.9**	39.8 ± 1.1**	
Betulinic acid (**4**)	0.0 ± 10	0.8 ± 6.7	1.6 ± 3.3	2.7 ± 2.3	24.0 ± 2.8*	
Pinoresinol (**7**)	0.0 ± 2.6	16.2 ± 1.1	50.6 ± 1.0**	95.7 ± 0.9**	100.0 ± 0.3**	27.9
Pinoresinol-4-*O*-glucoside (**8**)	0.0 ± 7.4	11.9 ± 5.5	3.9 ± 9.2	5.2 ± 7.6	27.8 ± 9.0*	
Epipinoresinol (**9**)	0.0 ± 7.3	17.6 ± 7.6	6.0 ± 2.5	5.4 ± 8.0	53.0 ± 3.7**	287
(–)-Phillygenin (**10**)	0.0 ± 2.6	2.1 ± 1.4	2.8 ± 4.8	29.3 ± 4.3**	80.9 ± 2.3**	152
Forsythenin (**11**)	0.0 ± 7.3	12.4 ± 8.1	5.3 ± 3.5	11.8 ± 5.2	9.3 ± 4.8	
( +)-Rengyolone (**13**)	0.0 ± 9.6	3.5 ± 8.1	16.4 ± 1.7	30.7 ± 4.2**	71.2 ± 0.8**	161
Forsythiaside A (**16**)	0.0 ± 1.5	36.8 ± 4.4**	58.5 ± 3.0**	86.9 ± 0.8**	95.8 ± 0.6**	18.6
Suspensaside mrthyl ether (**17**)	0.0 ± 7.5	24.4 ± 5.0**	34.2 ± 2.6**	58.2 ± 1.6**	59.7 ± 0.5**	91.5
Suspensaside A (**19**)	0.0 ± 7.5	32.4 ± 4.5**	44.5 ± 1.8**	73.3 ± 0.9**	86.9 ± 1.5**	31.1
Protocatechuic aldehyde (**20**)	0.0 ± 4.0	18.1 ± 6.2*	20.8 ± 3.2*	37.5 ± 7.5**	58.0 ± 4.0**	202
D-Saccharic acid 1,4-lactone	0.0 ± 2.0	22.8 ± 0.6**	46.4 ± 0.8**	70.7 ± 0.7**	86.6 ± 0.3**	38.6

Each value represents the mean ± S.E.M (*N* = 5)

Asterisks denote significant differences from the control group, *p < 0.05, **p < 0.01

To characterize the binding affinity between the inhibitor and enzyme, the kinetic parameter *K*i value and mode of inhibition of the most effective isolate phenylethanoid glycoside forsythiaside A (**16**) was characterized using Lineweaver–Burk plots. As shown in Fig. 4, **16** (*K*i = 11.6 μM) exhibited non-competitive inhibition, with an equivalent *K*i value but different inhibition type as d-saccharic acid 1,4-lactone (7.8 μM, competitive).

## Conclusion

In conclusion, the new diterpene rengyonoic acid (**1**) was isolated from the methanol extract of the fruit of *F. suspensa*. Among the isolates, **1** (IC_50_ = 117 μM), pinoresinol (**7**, 27.9 μM), forsythiaside A (**16**, 18.6 μM), and suspensaside A (**19**, 31.1 μM) exhibited β-glucuronidase inhibitory activity equivalent to that of the positive control d-saccharic 1,4-lactone (38.6 μM). To the best of our knowledge, this is the first report of β-glucuronidase inhibitory activity for pinoresinol (**7**), forsythiaside A (**16**), and suspensaside A (**19**). Considering both the inhibitory potency and the relative abundance of the constituents in the extract, phenylethanoid glycoside constituents, particularly forsythiaside A (**16**) and suspensaside A (**19**), are likely to be major contributors to the overall β-glucuronidase inhibitory activity of the extract. The detailed mechanisms of action and structure–activity relationships require further examination. In kinetic analysis, **16** (*K*i = 11.6 μM) exhibited non-competitive inhibition with an equivalent *K*i value but different inhibition type to those of d-saccharic acid 1,4-lactone (7.8 μM, competitive). The detailed structural features of phenylethanoids leading to β-glucuronidase inhibitory activity require further analysis.

## Materials and methods

### General

The following instruments were used to obtain physical data: specific rotations were obtained using a Horiba SEPA-300 digital polarimeter (*l* = 5 cm) (Kyoto, Japan); UV spectra were obtained using a Shimadzu UV-1600 spectrometer (Kyoto, Japan); IR spectra were obtained using a Shimadzu FTIR-8100 spectrometer; ^1^H NMR spectra were obtained using Ascend Evo 800 (800 MHz, Bruker Corporation, Billerica, MA, USA), JNM-ECA800 (800 MHz), JNM-ECS400, and JNM-AL400 (400 MHz) (JEOL Ltd., Tokyo, Japan) spectrometers; ^13^C NMR spectra were obtained using Ascend Evo 800 (200 MHz), JNM-ECA800 (200 MHz), JNM-ECS400, and JNM-AL400 (100 MHz) spectrometers with tetramethylsilane as an internal standard; ESIMS and high-resolution ESIMS data were obtained using a Exactive Plus mass spectrometer (Thermo Fisher Scientific, Waltham, MA, USA); the HPLC detector used was a Shimadzu SPD-10A UV–VIS detector (detection: 230 nm); the HPLC columns used were a Cosmosil 5C_18_-MS-II (Nacalai Tesque, Kyoto, Japan), 4.6 mm i.d. × 250 mm and 20 mm i.d. × 250 mm for analytical and preparative purposes, respectively.

The following experimental conditions were used for CC: highly porous synthetic resin, Diaion HP-20 (Mitsubishi Chemical Co., Tokyo, Japan); normal-phase silica gel CC, silica gel 60N (Kanto Chemical Co., Ltd., Tokyo, Japan; 63–210 mesh, spherical, neutral); reversed-phase ODS CC, Chromatorex ODS DM1020T (Fuji Silysia Chemical, Ltd., Aichi, Japan; 100–200 mesh); thin-layer chromatography (TLC), pre-coated TLC plates with silica gel 60F_254_ (Merck, Darmstadt, Germany, 0.25 mm) (normal-phase) and silica gel RP-18 WF_254S_ (Merck, 0.25 mm) (reversed-phase); reversed-phase HPTLC, pre-coated TLC plates with silica gel RP-18 WF_254S_ (Merck, 0.25 mm); detection was performed by spraying 1% Ce(SO_4_)_2_–10% aqueous H_2_SO_4_, followed by heating.

### Plant material

The fruit of *F. suspensa* were collected in Shanxi province, China, as described previously [96]. Identification of the plant material was confirmed by the Garden of Medicinal Plants, Kindai University and by one of the authors (T.M.). A voucher specimen (lot. no. K083A7) of this plant material is on file in our laboratory.

### Extraction and isolation

Dried fruit of *F. suspensa* (2000 g) were extracted three times with MeOH under reflux for 3 h. Evaporation of the combined extracts under reduced pressure yielded the MeOH extract (274.6 g, 13.6% from the dried material). An aliquot (241.1 g) of the MeOH extract was partitioned in an EtOAc-H_2_O (1:1, v/v) mixture, yielding an EtOAc-soluble fraction (125.1 g, 7.07%) and an aqueous phase. The aqueous phase was subjected to Diaion HP-20 CC (3.0 kg, H_2_O → MeOH), which yielded H_2_O-eluted (8.9 g, 0.50%) and MeOH-eluted (67.4 g, 3.81%) fractions. An aliquot (110.3 g) of the EtOAc-soluble fraction was subjected to normal-phase silica gel CC [3.0 kg, *n*-hexane–EtOAc (30:1 → 10:1 → 5:1 → 2:1 → 1:1, v/v) → EtOAc → MeOH], which yielded nine fractions {Fr. 1 (0.36 g), Fr. 2 (6.07 g), Fr. 3 (3.81 g), Fr. 4 (12.03 g), Fr. 5 [= betulinic acid (**4**, 10.36 g, 0.66%)], Fr. 6 (25.65 g), Fr. 7 (9.65 g), Fr. 8 (4.133 g), and Fr. 9 (41.48 g)}. Fraction 7 (9.65 g) was subjected to reversed-phase ODS CC [300 g, MeOH–H_2_O (50:50 → 70:30 → 90:10, v/v)] to afford seven fractions [Fr. 7–1 (620.0 mg), Fr. 7–2 (1.20 g), Fr. 7–3 (240.0 mg), Fr. 7–4 (1.65 g), Fr. 7–5 (110.0 mg), Fr. 7–6 (530.0 mg), and Fr. 7–7 (4.64 g)]. Fraction 7–1 (620.0 mg) was purified using HPLC [column: Cosmosil 5C_18_-MS-II, detection: UV (254 nm), mobile phase: MeOH–1% aqueous H_2_O (60:40, v/v)] to give salicifoliol (**12**, 4.6 mg, 0.0003%). Fraction 7–2 (600.0 mg) was purified using HPLC [column: Cosmosil 5C_18_-MS-II, detection: UV (254 nm), mobile phase: MeOH–1% aqueous H_2_O (70:30, v/v)] to give epipinoresinol (**9**, 29.6 mg, 0.0038%). Fraction 7–4 (300.0 mg) was purified using HPLC [column: Cosmosil 5C_18_-MS-II, detection: UV (254 nm), mobile phase: MeOH-1% aqueous H_2_O (50:50, v/v)] to give phillygenin (**10**, 54 mg, 0.0019%). Fraction 7–5 (110.0 mg) was purified using HPLC [column: Cosmosil 5C_18_-MS-II, detection: UV (254 nm), mobile phase: MeOH–1% aqueous H_2_O (50:50, v/v)] to give **10** (3.6 mg, 0.00023%). Fraction 7–7 (100.0 mg) was purified using HPLC [column: Cosmosil 5C_18_-MS-II, detection: UV (254 nm), mobile phase: MeOH–1% aqueous H_2_O (80:20, v/v)] to obtain 3β-hydroxyanticopalic (**2**, 4.4 mg, 0.00028%). Fraction 8 (4.13 g) was subjected to reversed-phase ODS CC [120 g, MeOH–H_2_O (50:50 → 80:20, v/v) → MeOH] to afford 11 fractions [Fr. 8–1 (308.7 mg), Fr. 8–2 (559.9 mg), Fr. 8–3 (617.5 mg), Fr. 8–4 (113.9 mg), Fr. 8–5 (166.3 mg), Fr. 8–6 (295.2 mg), Fr. 8–7 (1.65 g), Fr. 8–8 (96.6 mg), Fr. 8–9 (140.4 mg), Fr. 8–10 (186.0 mg), and Fr. 8–11 (202.0 mg)]. Fraction 8–1 (308.7 mg) was purified using HPLC [Cosmosil 5C_18_-MS-II, UV (230 nm), MeOH–1% aqueous AcOH (20:80, v/v)] to give (+)-rengyolone (**13**, 9.5 mg, 0.00061%) and protocatechuic aldehyde (**20**, 9.7 mg, 0.00062%). Fraction 8–2 (559.9 mg) was purified using HPLC [Cosmosil 5C_18_-MS-II, UV (230 nm), MeOH–1% aqueous AcOH (45:55, v/v)] to give pinoresinol (**7**, 232.2 mg, 0.016%) and forsythienin (**11**, 7.9 mg, 0.00058%). Fraction 8–5 (166.3 mg) was purified using HPLC [Cosmosil 5C_18_-MS-II, UV (230 nm), MeOH–1% aqueous AcOH (65:35, v/v)] to give forsyditerpene A (**3**, 10.1 mg, 0.00065%). Fraction 8–6 (295.2 mg) was purified using HPLC [Cosmosil 5C_18_-MS-II, UV (230 nm), MeOH–1% aqueous AcOH (50:50, v/v) and CH_3_CN–1% aqueous AcOH (50:50, v/v)] to give rengyonoic acid (**1**, 10.0 mg, 0.00064%) and isomucronulatol (**15**, 2.0 mg, 0.00013%). Fraction 8–7 (1.65 g) was purified using HPLC [Cosmosil 5C_18_-MS-II, UV (230 nm), CH_3_CN–1% aqueous AcOH (20:80, v/v)] to give hederagenic acid (**5**, 32.6 mg, 0.0021%) and 23-hydroxyursolic acid (**6**, 40.0 mg, 0.0026%). An aliquot (50.0 g) of the MeOH-eluted fraction was subjected to normal-phase silica gel CC [2.0 kg, CHCl_3_–MeOH–H_2_O (30:3:0.3 → 20:3:0.3 → 10:3:0.3 → 6:4:1, v/v) → MeOH] to yield 12 fractions [Fr. 1 (0.46 g), Fr. 2 (2.55 g), Fr. 3 (2.02 g), Fr. 4 (2.60 g), Fr. 6 (3.02 g), Fr. 7 (5.08 g), Fr. 8 (4.28 g), Fr. 9 (3.55 g), Fr. 10 (1.94 g), Fr. 11 (4.73 g) and Fr. 12 (0.71 g)]. Fraction 4 (2.60 g) was subjected to reversed-phase ODS CC [80 g, MeOH–H_2_O (30:70 → 40:60 → 60:40, v/v) → MeOH], which yielded eight fractions [Fr. 4–1 (173.8 mg), Fr. 4–2 (220.1 mg), Fr. 4–3 (99.8 mg), Fr. 4–4 (255.3 mg), Fr. 4–5 (98.7 mg), Fr. 4–6 (271.9 mg), Fr. 4–7 (681.1 mg) and Fr. 4–8 (454.0 mg)]. Fraction 4–6 (271.8 mg) was purified using HPLC [Cosmosil 5C_18_-MS-II, UV (254 nm), MeOH–1% aqueous AcOH (30:70, v/v)] to give pinoresinol-4-*O*-glucoside (**8**, 20.9 mg, 0.0016%). Fraction 6 (3.02 g) was subjected to reversed-phase ODS CC [90 g, MeOH–H_2_O (30:70 → 40:60 → 50:50, v/v) → MeOH], which yielded nine fractions [Fr. 6–1 (148.4 mg), Fr. 6–2 (88.2 mg), Fr. 6–3 (591.2 mg), Fr. 6–4 (253.3 mg), Fr. 6–5 (716.9 mg), Fr. 6–6 (786.4 mg), Fr. 6–7 (163.1 mg), Fr. 6–8 (251.8 mg), and Fr. 6–9 (111.1 mg)]. Fraction 6–3 (591.2 mg) was purified using HPLC [Cosmosil 5C_18_-MS-II, UV (254 nm), MeOH–1% aqueous AcOH (30:70, v/v)] to give forsythiaside A (**16**, 75.0 mg, 0.0057%). Fraction 7 (5.08 g) was subjected to reversed-phase ODS CC [150 g, MeOH–H_2_O (30:70 → 40:60 → 50:50, v/v) → MeOH], which yielded 11 fractions {Fr. 7–1 (191.9 mg), Fr. 7–2 (88.4 mg), Fr. 7–3 (80.5 mg), Fr. 7–4 (193.6 mg), Fr. 7–5 (238.9 mg), Fr. 7–6 (657.2 mg), Fr. 7–7 (1494.0 mg), Fr. 7–8 [= rutin (**14**, 66.0 mg, 0.0050%)], Fr. 7–9 (428.9 mg), Fr. 7–10 (66.6 mg), and Fr. 7–11 (23.8 mg)}. Fraction 7–4 (193.6 mg) was purified using HPLC [Cosmosil 5C_18_-MS-II, detection: UV (210 nm), MeOH–1% aqueous AcOH (10:90, v/v)] to give suspensaside C (**18**, 18.2 mg, 0.0014%). Fraction 7–7 (1494.0 mg) was purified using HPLC [Cosmosil 5C_18_-MS-II, UV (210 nm), MeOH–1% aqueous AcOH (35:65, v/v)] to give suspensaside methyl ether (**17**, 9.6 mg, 0.00073%) and suspensaside A (**19**, 82.2 mg, 0.0063%). The isolated compounds (**2–20**) were unambiguously identified by comparing their physical and spectroscopic data with those of commercially available samples.

### Rengyonoic acid (**1**)

White powder, [α]_D_^23^ + 25.2 (*c* 0.29, MeOH); UV [MeOH, nm (long ε)]: 216 (3.80); IR (KBr) *v*_max_: 3400, 1695, 1645, 1450, 1240 cm^−1^; ECD [nm (Δε), MeOH]: 219 (+ 2.17) (Figs. 3); ^1^H (800 MHz, pyridine-*d*_5_) and ^13^C NMR (200 MHz, pyridine-*d*_5_): data are shown in Table 2 (Figures S1 and S2); the ^1^H–^1^H COSY, HSQC, HMBC, and pNOESY spectra (pyridine-*d*_5_) are provided in the Supporting Information (Figures S3–S6); ^1^H (800 MHz, DMSO-*d*_6_) and ^13^C NMR (200 MHz, DMSO-*d*_6_): data shown in Table 2 (Figures S7 and S8); ^1^H–^1^H COSY, HMQC, HMBC, and pNOESY spectra (DMSO-*d*_6_) are provided in the Supporting Information (Figures S9–S12); positive-ion ESI–MS *m/z*: 359 [M + Na]^+^; high-resolution ESIMS *m/z*: 359.2186 [M + Na]^+^ (calcd for C_20_H_32_O_4_Na, 359.2193) (Figure S13).

### Calculation of theoretical ECD spectra of **1**

The initial geometry of the possible conformers of **1** [(4*S*,5*R*,6*R*,9*S*,10*R*)- and (4*R*,5*S*,6*S*,9*R*,10*S*)-forms] were generated and geometrically optimized in vacuum using the Merck molecular force field (MMFF) as implemented in the Spartan'24 program (Wavefunction, Inc., Irvine, CA, USA, https://www.wavefun.com/). Stable energy conformers with Boltzmann distributions greater than 1% were further optimized at the ωB97X-D/def2-TZVP level of density functional theory (DFT). To confirm that none of the conformers showed imaginary frequencies and to obtain the enthalpies (*H*), including the zero-point energy (ZPE) correction, normal-mode analysis was performed at the same level [97, 98]. The distinctive low-energy conformers for **1** with Boltzmann distributions greater than 1% were subjected to ECD calculations using time-dependent DFT (TD-DFT) at the ωB97X-D/ma-TZVPP (def2-TZVPP basis set with the s and p diffuse basis functions on non-hydrogen atoms) level [38, 99–101]. All DFT and TD-DFT calculations were performed using an integral equation formalism polarizable continuum model (IEFPCM) in MeOH with Gaussian 16 [102] (https://gaussian.com/citation_a03/). The resulting rotatory strengths of the lowest 30 excited states for each conformer were converted into Gaussian-type curves with half bands using SpecDis v1.71 [103]. The calculated ECD spectra were composed after correction based on the Boltzmann distribution of the conformers and their relative enthalpies, including the ZPE correction (Δ*H*). The low-energy conformers of **1** used for the TD-DFT calculations are shown in Figures S14.

### Bioassay

#### β-Glucuronidase inhibitory activity

A solution of β-glucuronidase (from *E. coli*, Sigma-Aldrich, St. Louis, MO, USA) was prepared to a working concentration of 5 U/mL by dissolving the enzyme in 200 mM phosphate buffer (pH 6.0). The substrate, 4-nitrophenyl β-d-glucuronide (Tokyo Chemical Industry Co., Ltd., Tokyo, Japan), was dissolved in the buffer to a final assay concentration of 250 μM. All enzyme and substrate solutions were freshly prepared prior to use and kept on ice until the assay. β-Glucuronidase inhibitory activity was determined as previously reported [70] with slight modifications. Briefly, the assay was performed in 96-well microplates. β-Glucuronidase enzyme solution (100 μL) or blank buffer was preincubated with sample solution (20 μL) and 30 μL of the buffer at 37 °C for 20 min. The substrate solution (50 μL) was then added, and the mixture was incubated at 37 °C for 60 min. After incubation, the optical density (O.D.) was measured using a microplate reader (SH-9000, CORONA ELECTRIC Co., Ltd., Ibaraki, Japan) at 405 nm with a reference wavelength of 670 nm. The final concentration of dimethyl sulfoxide (DMSO) in the test solution was 1.0%, and DMSO did not appear to influence the inhibitory activity. d-Saccharic acid 1,4-lactone (FUJIFILM Wako Pure Chemical Industries Ltd., Osaka, Japan) was used as a reference compound [94, 95]. All experiments were performed in quintuplicate, and IC_50_ values were determined graphically.

#### Kinetic analysis

The enzyme and test samples (10, 25, and 50 μM) were incubated with increasing concentrations of substrate (0.1, 0.125, 0.25, and 0.5 mM). The mode of inhibition was analyzed using the Lineweaver–Burk plot, with the inverse reaction velocity of the substrate plotted on the vertical axis and inverse velocity of the final concentration of the substrate on the horizontal axis, with and without the test sample.

#### Statistics

Values are expressed as the mean ± S.E.M. One-way analysis of variance followed by Dunnett's test was used for statistical analysis. Probability (*p*) values less than 0.05 were considered significant.

## Supplementary Information

Below is the link to the electronic supplementary material.

Supplementary file1 (PDF 14805 KB)
