# Supplementary material for: β-glucuronidase inhibitors isolated from the fruit of Forsythia suspensa (Thunb.) Vahl
Source: J Nat Med. 2026 Jul 28;80(5):1508–20. doi: 10.1007/s11418-026-02070-1 (PMC13547174; doi:10.1007/s11418-026-02070-1)

Supplementary Information

β-Glucuronidase inhibitors isolated from the fruit of *Forsythia suspensa* (Thunb.) Vahl

Shogo Nishikawa[1,3] · Ryuya Takada[1,3] · Yusuke Shimomoto[1] · Sara Morishita[1] · Yoshiaki Manse[1] · Tomoe Ohta[4] · Tatsusada Yoshida[5] · Takahiro Matsumoto[4] · Toshio Morikawa[1,2,3]*

[1] Pharmaceutical Research and Technology Institute, Kindai University, 3-4-1 Kowakae, Higashi-osaka, Osaka 577-8502, Japan
[2] Antiaging Center, Kindai University, 3-4-1 Kowakae, Higashi-osaka, Osaka 577-8502, Japan
[3] Graduate School of Pharmacy, Kindai University, 3-4-1 Kowakae, Higashi-osaka, Osaka 577-8502, Japan
[4] Kyoto Pharmaceutical University, Misasagi, Yamashina-ku, Kyoto 607-8412, Japan
[5] Faculty of Pharmaceutical Sciences, Nagasaki International University, 2825-7 Huis Ten Bosch Machi, Sasebo, Nagasaki 859-3298, Japan

* Correspondence: morikawa@kindai.ac.jp; Tel.: +81-6-4307-4306

Figure S1. ^{1}H-NMR spectrum of rengyonoic acid (**1**) in pyridine-d_5 ········ S1
Figure S2. ^{13}C NMR and DEPT135 spectra of **1** in pyridine-d_5 ········ S2
Figure S3. DQF-COSY spectrum of **1** in pyridine-d_5 ········ S3
Figure S4. HSQC spectrum of **1** in pyridine-d_5 ········ S4
Figure S5. HMBC spectrum of **1** in pyridine-d_5 ········ S5
Figure S6. Phase-sensitive pNOESY spectrum of **1** in pyridine-d_5 ········ S6
Figure S7. ^{1}H-NMR spectrum of **1** in CD_3OD ········ S7
Figure S8. ^{13}C NMR and DEPT135 spectra of **1** in CD_3OD ········ S8
Figure S9. DQF-COSY spectrum of **1** in CD_3OD ········ S9
Figure S10. HSQC spectrum of **1** n CD_3OD ········ S10
Figure S11. HMBC spectrum of **1** n CD_3OD ········ S11
Figure S12. Phase-sensitive pNOESY spectrum of **1** n CD_3OD ········ S12
Figure S13. Positive-ion HRESIMS spectrum of **1** ········ S13
Figure S14. Optimized geometries, the minimum value of frequency, relative enthalpies including the ZPE correction, and Boltzmann distributions of conformers of rengyonoic acid (**1**) ········ S14

rengyonoic acid (1)

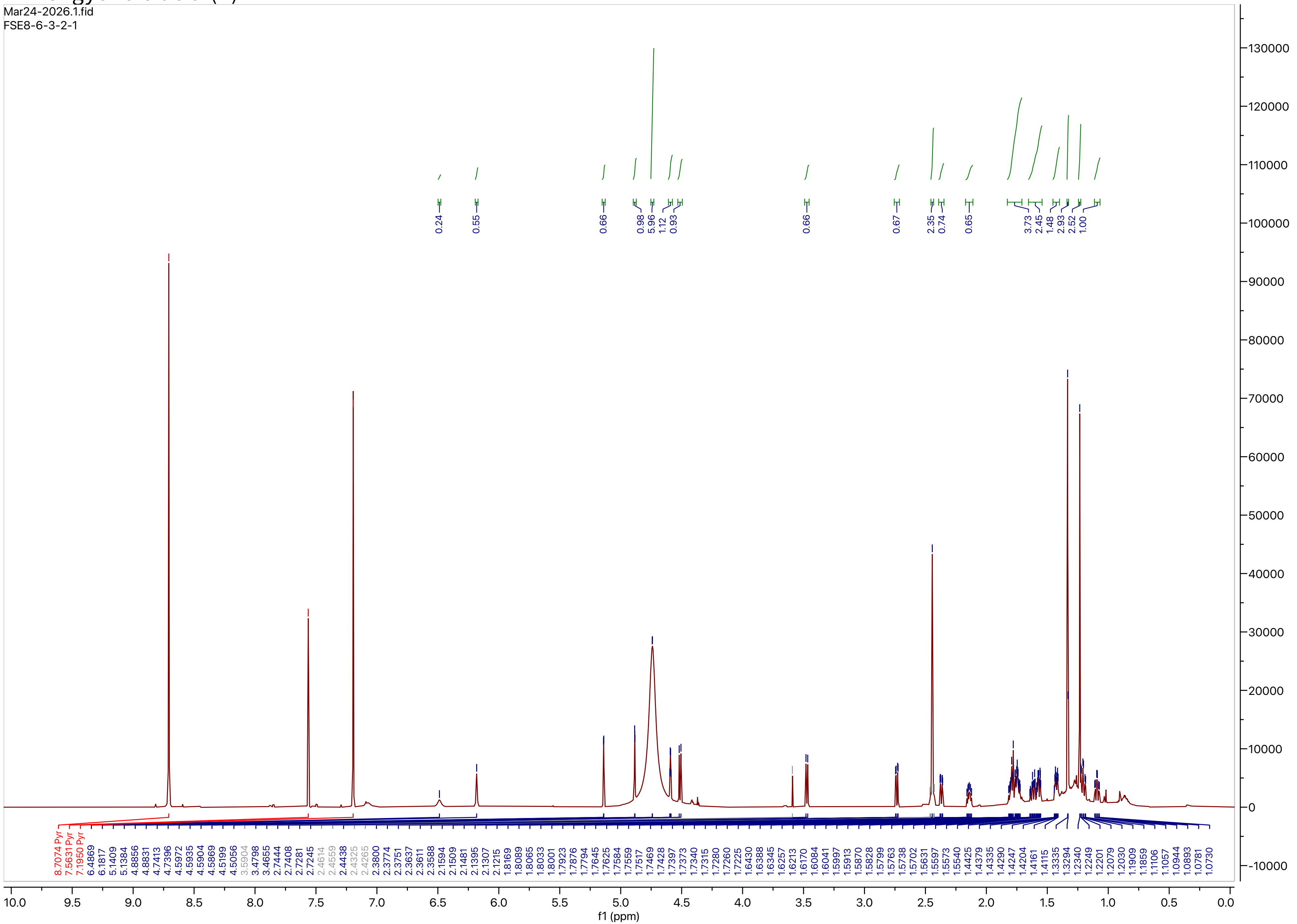

rengyonoic acid (1)

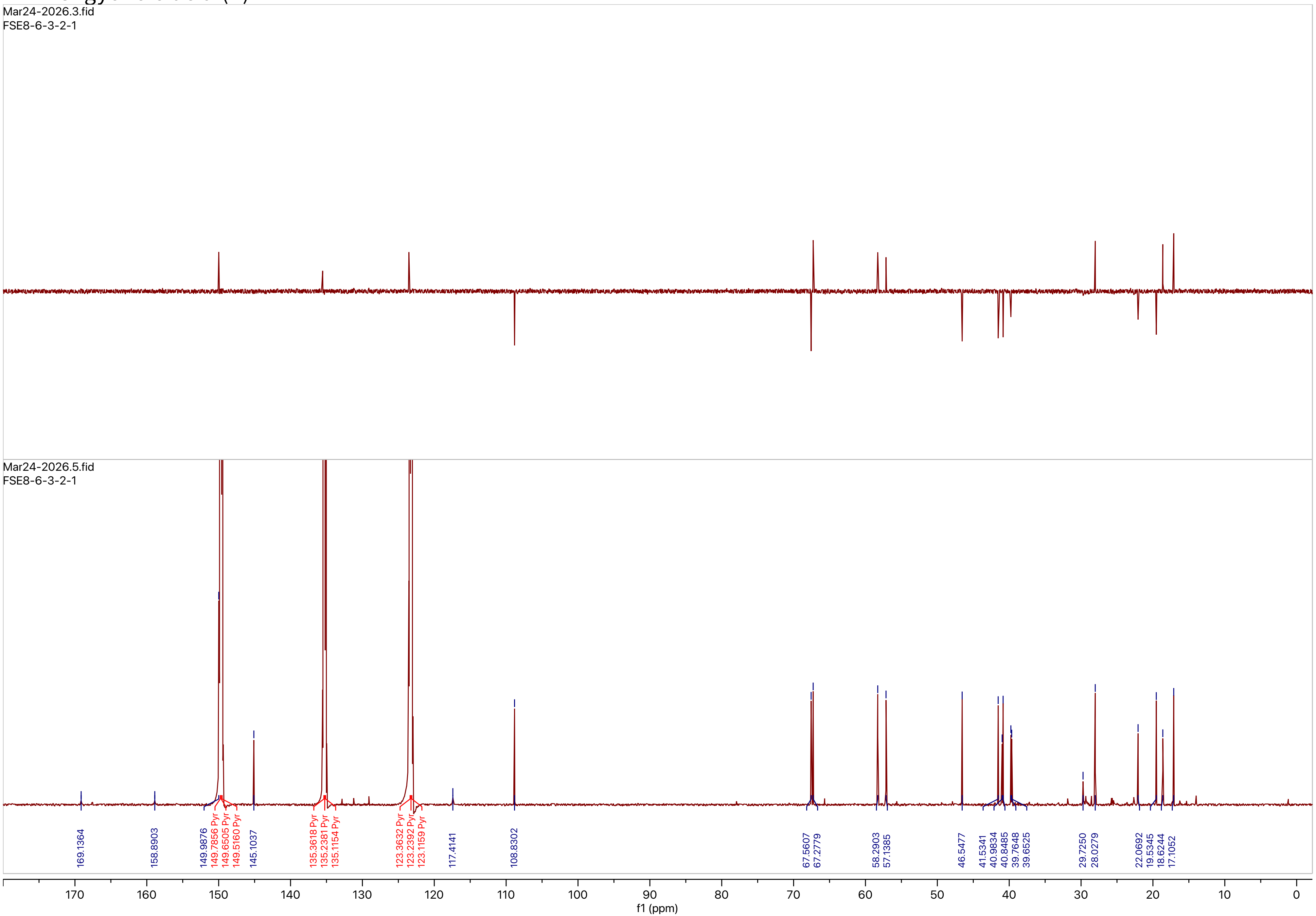

rengyonoic acid (1)

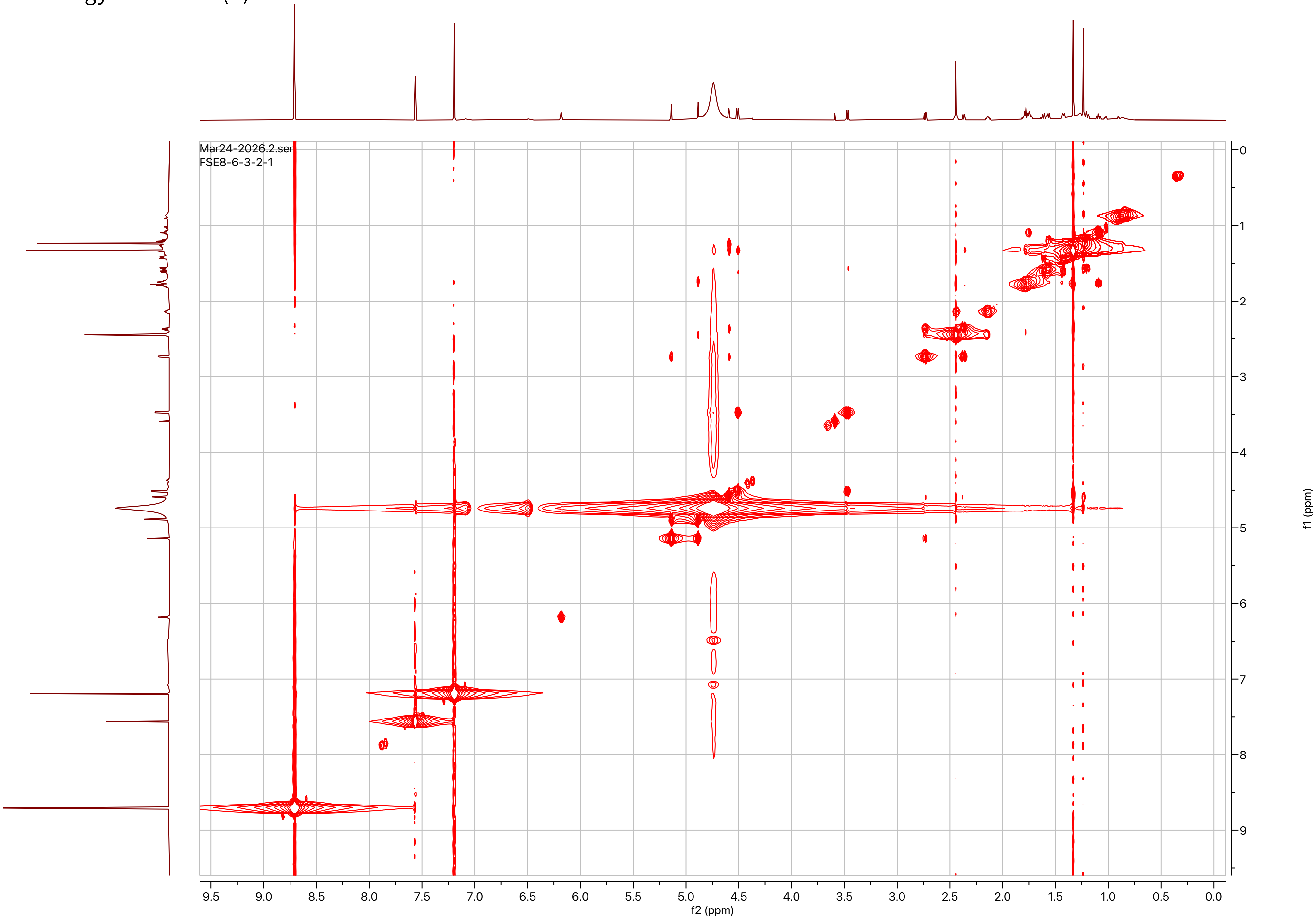

rengyonoic acid (1)

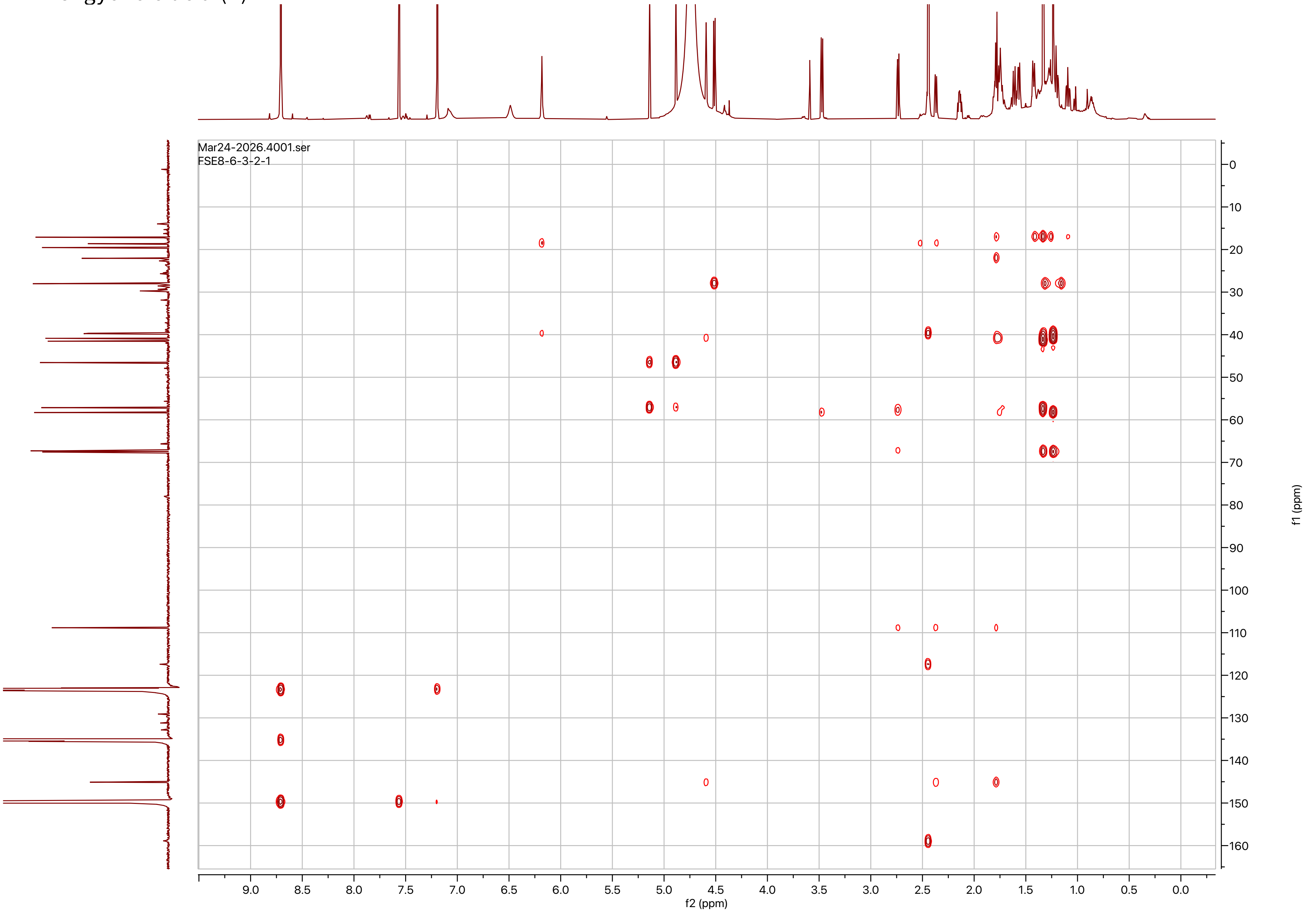

rengyonoic acid (1)

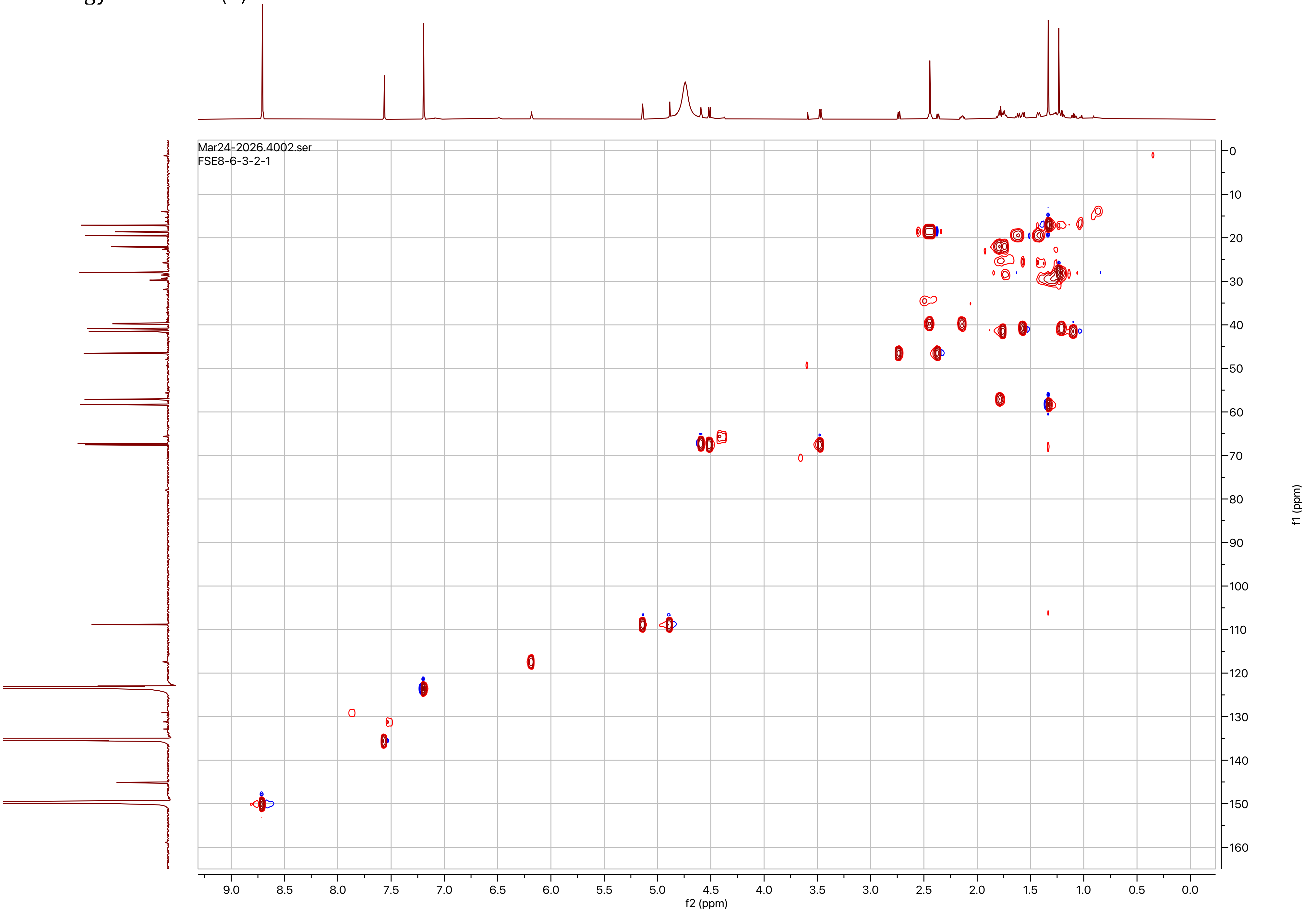

rengyonoic acid (1)

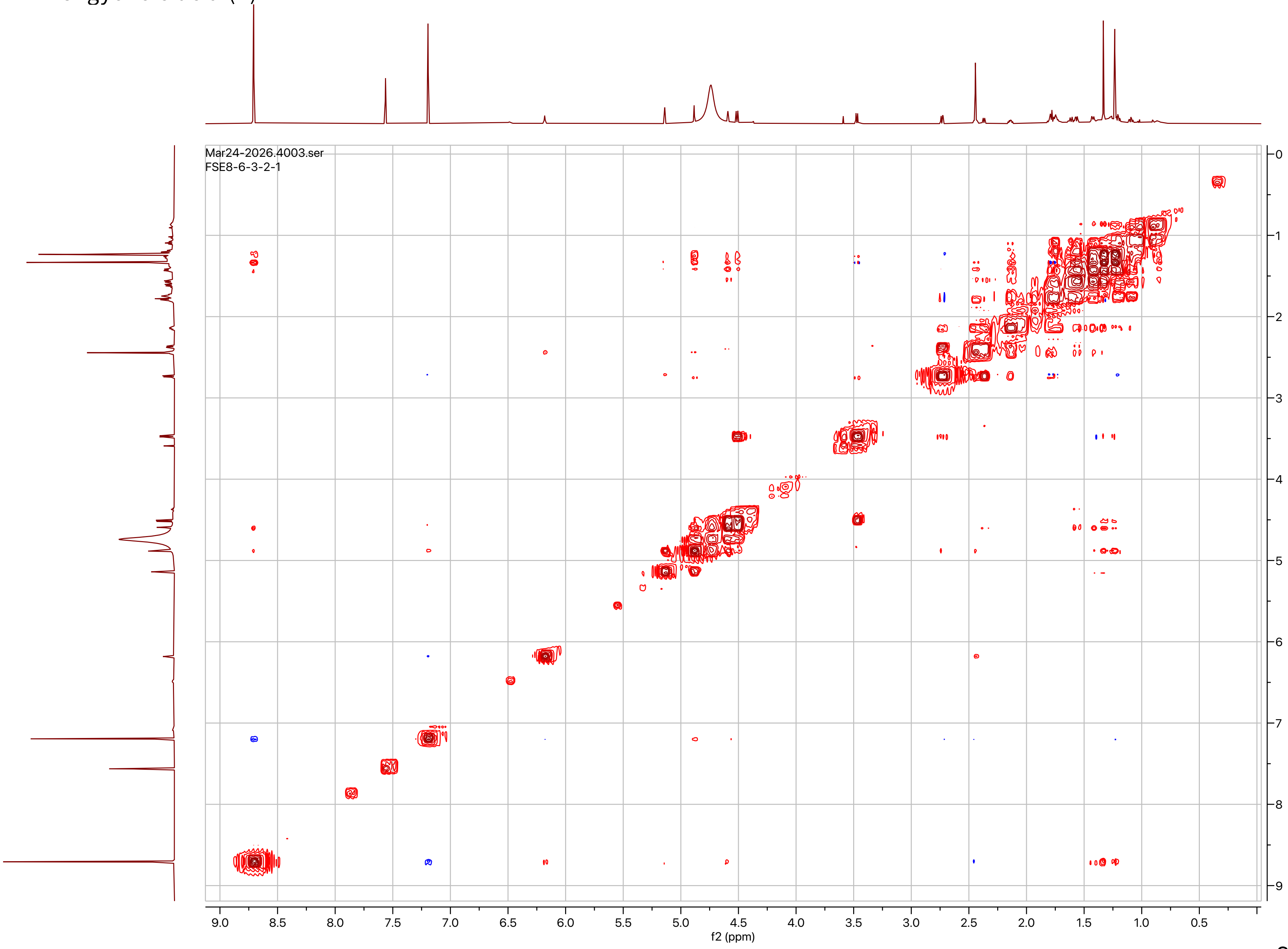

rengyonoic acid (1)

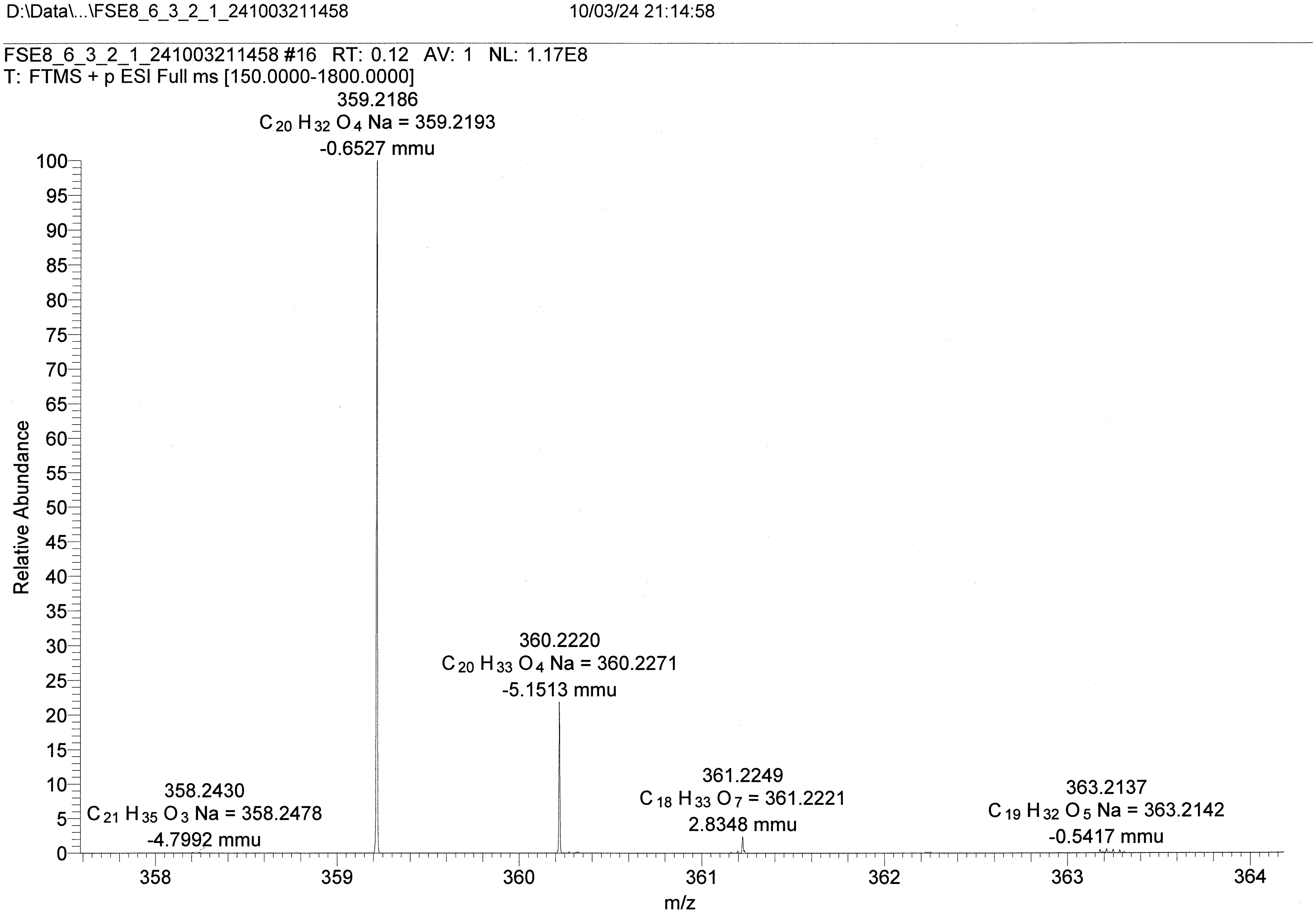

rengyonoic acid (1)

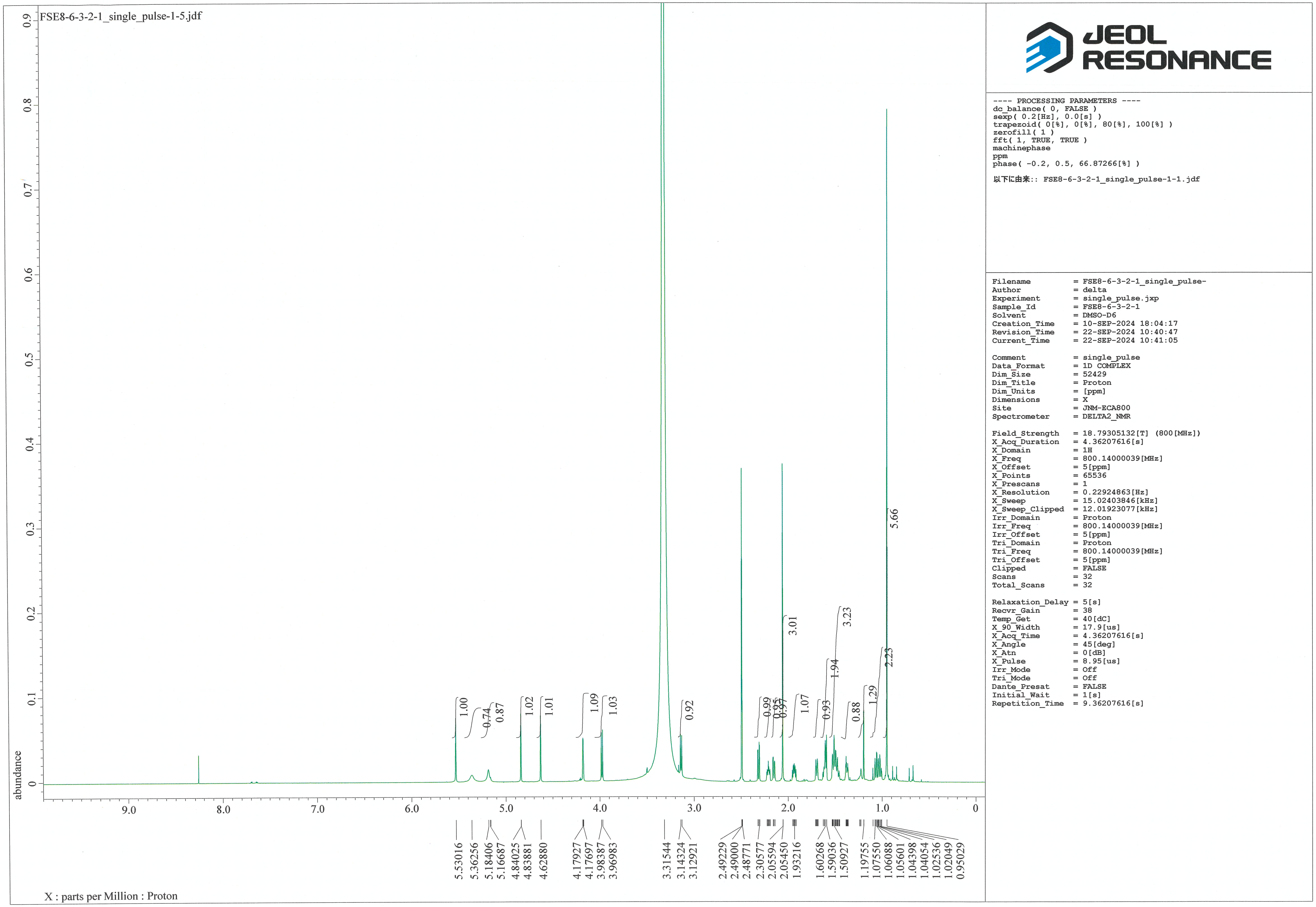

rengyonoic acid (1)

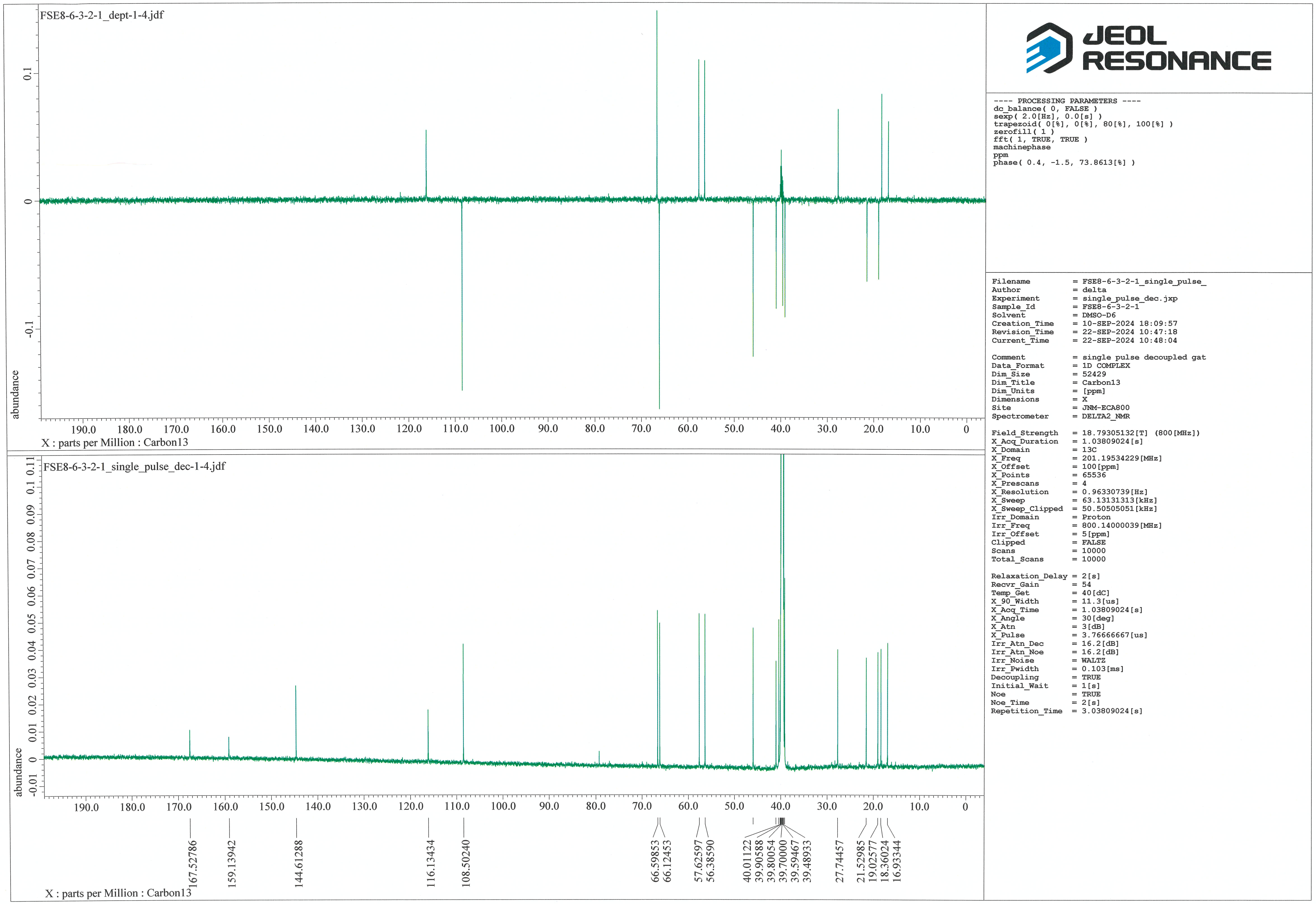

rengyonoic acid (1)

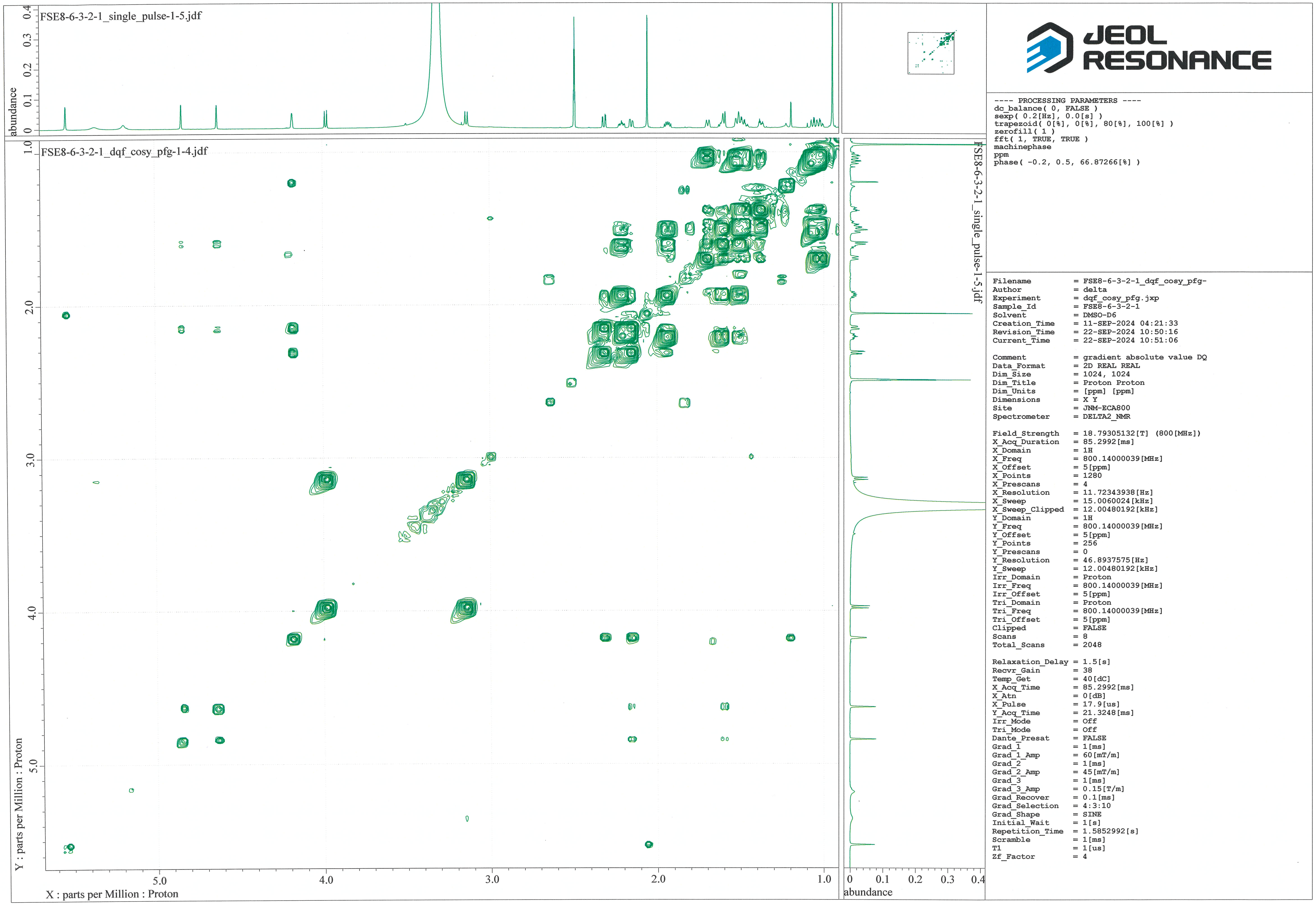

rengyonoic acid (1)

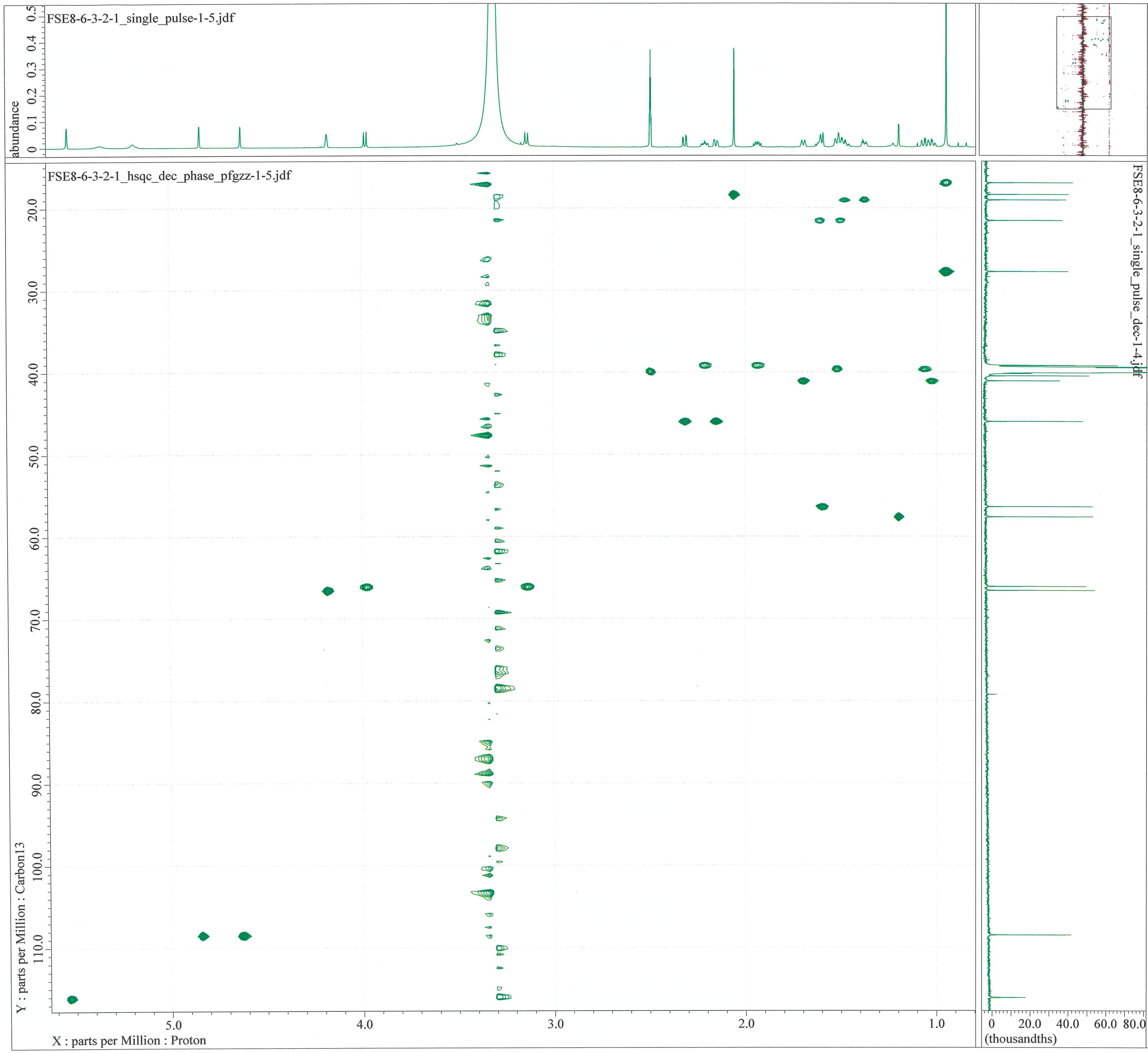


```
---- PROCESSING PARAMETERS ----
dc_balance( 0, FALSE )
sexp( 0.2[Hz], 0.0[s] )
trapezoid( 0[%], 0[%], 80[%], 100[%] )
zerofill( 1 )
fft( 1, TRUE, TRUE )
machinephase
ppm
phase( -0.2, 0.5, 66.87266[%] )
```

```
Filename           = FSE8-6-3-2-1_hsqc_dec_phas
Author             = delta
Experiment         = hsqc_dec_phase_pfgzz.jxp
Sample_Id          = FSE8-6-3-2-1
Solvent            = DMSO-D6
Creation_Time      = 11-SEP-2024 05:16:51
Revision_Time      = 22-SEP-2024 10:52:35
Current_Time       = 22-SEP-2024 10:53:11

Comment            = absorption_hsqc
Data_Format        = 2D COMPLEX COMPLEX
Dim_Size           = 512, 819
Dim_Title          = Carbon13 Proton
Dim_Units          = [ppm] [ppm]
Dimensions         = X Y
Site               = JNM-ECA800
Spectrometer       = DELTA2_NMR

Field_Strength     = 18.79305132[T] (800[MHz])
X_Acq_Duration     = 68.23936[ms]
X_Domain           = 1H
X_Freq             = 800.14000039[MHz]
X_Offset           = 5[ppm]
X_Points           = 1024
X_Prescans         = 4
X_Resolution       = 14.65429922[Hz]
X_Sweep            = 15.0060024[kHz]
X_Sweep_Clipped    = 12.00480192[kHz]
Y_Domain           = 13C
Y_Freq             = 201.19534229[MHz]
Y_Offset           = 85.0[ppm]
Y_Points           = 256
Y_Prescans         = 0
Y_Resolution       = 133.77568493[Hz]
Y_Sweep            = 34.24657534[kHz]
Tri_Domain         = Proton
Tri_Freq           = 800.14000039[MHz]
Tri_Offset         = 5[ppm]
Clipped            = FALSE
Scans              = 8
Total_Scans        = 2048

Relaxation_Delay   = 1.5[s]
Recvr_Gain         = 38
Temp_Get           = 40[dC]
X_Acq_Time         = 68.23936[ms]
X_Atn              = 0[dB]
X_Pulse            = 17.9[us]
Y_Acq_Time         = 7.4752[ms]
Y_Atn              = 3[dB]
Y_P1_Correction    = 720
Y_Pulse            = 11.3[us]
Irr_Atn_Dec        = 20[dB]
Irr_Noise          = MPF8
Irr_Pwidth         = 80[us]
Tri_Mode           = Off
Composite_Pulse    = FALSE
Dante_Presat       = FALSE
Decoupling         = TRUE
Grad_1_Amp         = 0.105[T/m]
Grad_2_Amp         = 30[mT/m]
Initial_Wait       = 1[s]
J_Constant         = 140[Hz]
Phase_Qd           = {{0, 180}, {90, 270}}
Purge              = 0[ms]
Repetition_Time    = 1.56823936[s]
T1                 = 4.068[us]
T1_Cal1            = -17.832[us]
T1_Cal2            = -10.532[us]
T1_Cal3            = -3.232[us]
T1_Cal4            = 4.068[us]
T1_Cal5            = 11.368[us]
```

rengyonoic acid (1)

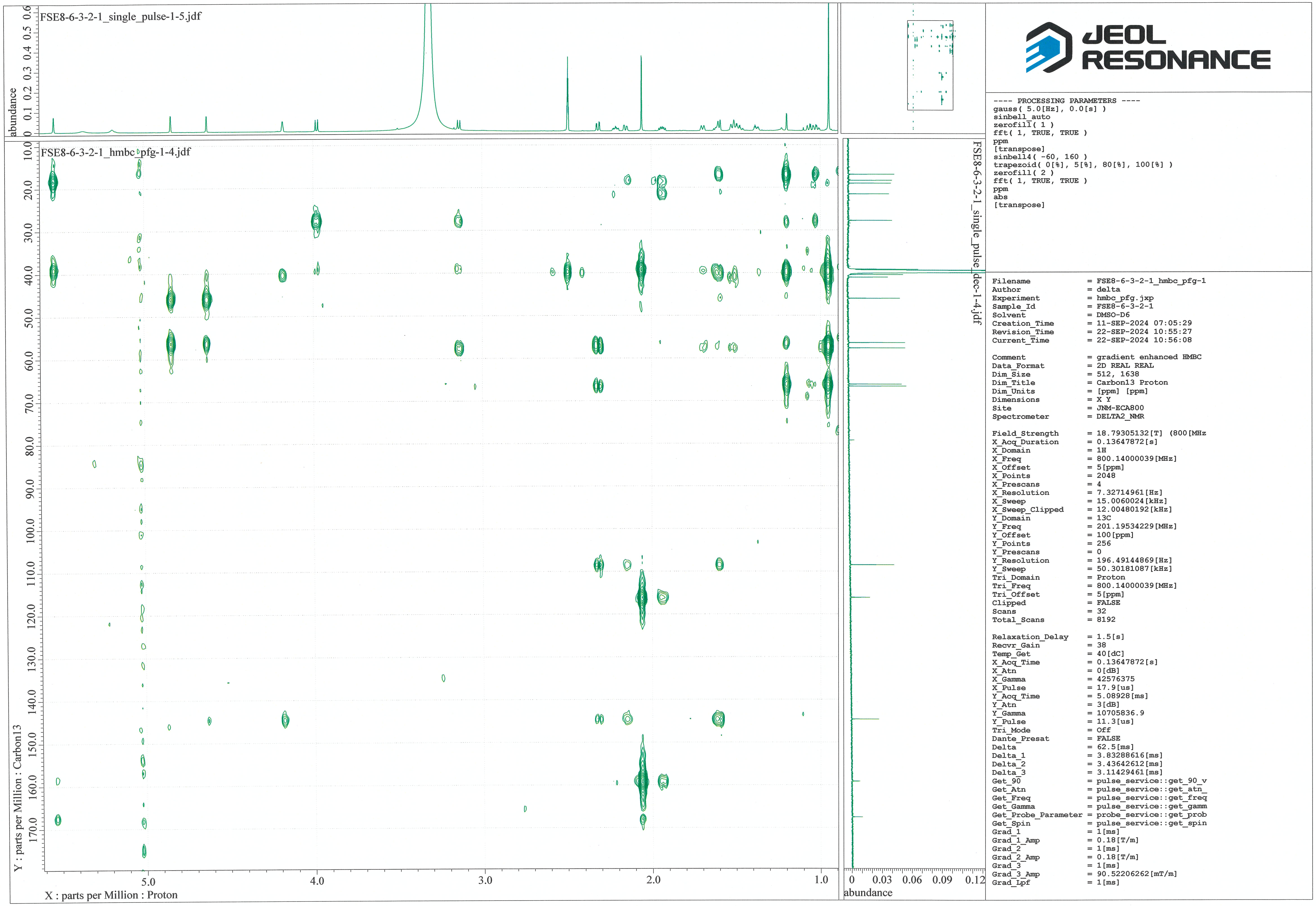

rengyonoic acid (1)

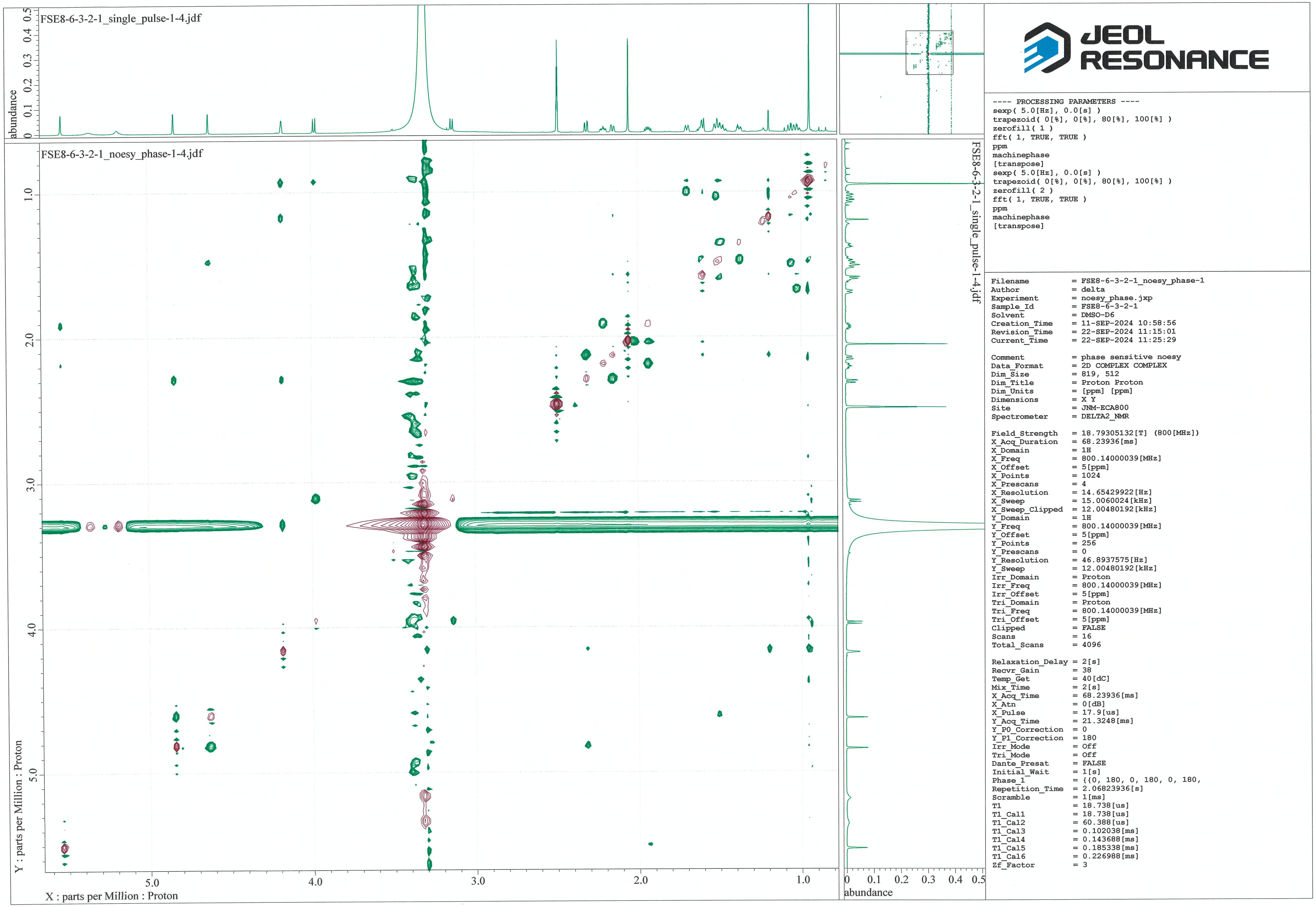

Figure S14. Optimized geometries, the minimum value of frequency, relative enthalpies including the ZPE correction, and Boltzmann distributions of conformers of rengyonoic acid (**1**).

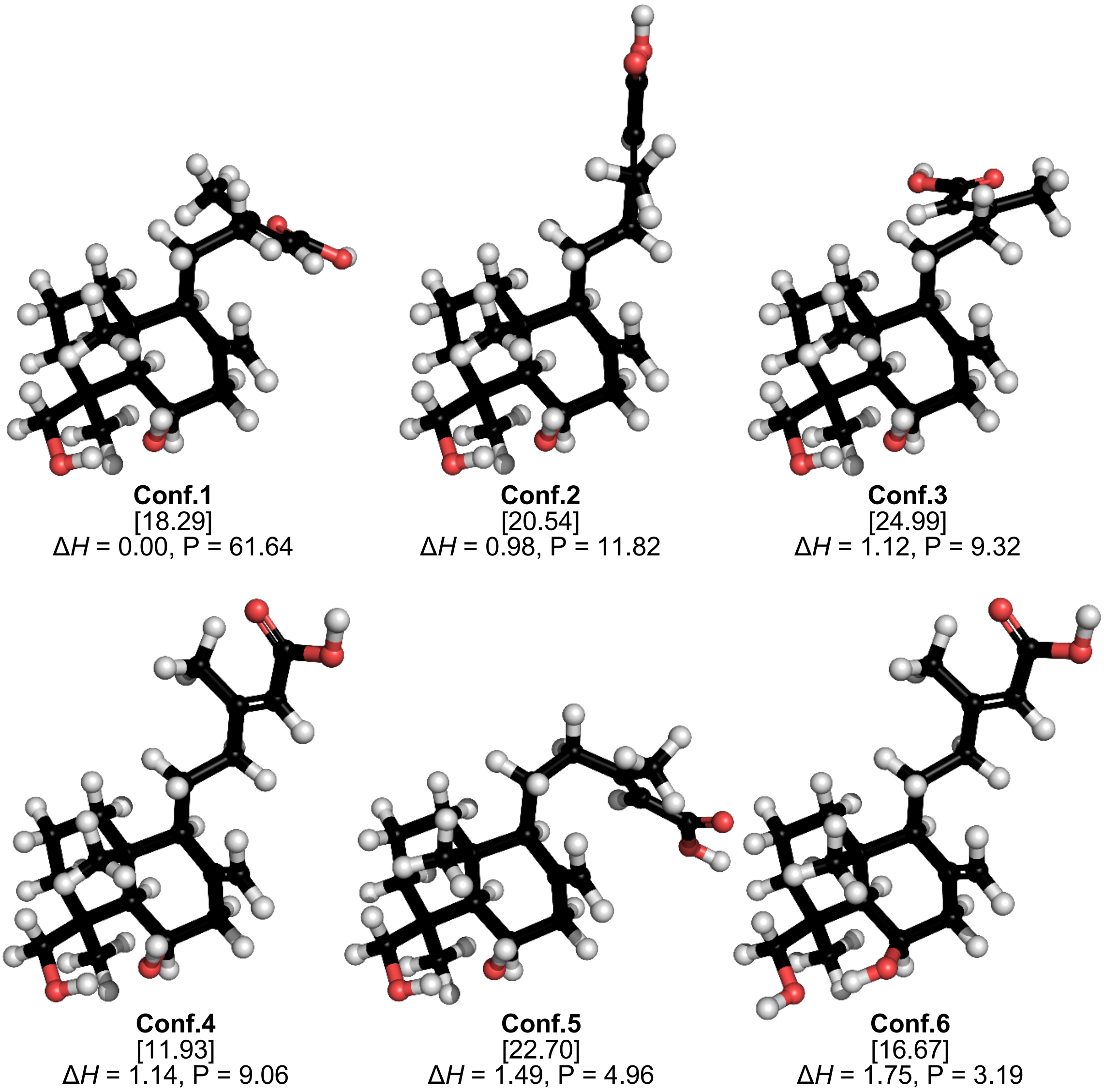

Supplement: Supplementary file 1 [file 11418_2026_2070_MOESM1_ESM.pdf]
